# Benchmarking Catalysts for Formic Acid/Formate Electrooxidation

**DOI:** 10.3390/molecules26164756

**Published:** 2021-08-06

**Authors:** Scott J. Folkman, Jesús González-Cobos, Stefano Giancola, Irene Sánchez-Molina, José Ramón Galán-Mascarós

**Affiliations:** 1 Institute of Chemical Research of Catalonia (ICIQ), The Barcelona Institute of Science and Technology (BIST), Av. Paisos Catalans, 16, 43007 Tarragona, Spain; sgiancola@iciq.es (S.G.); isanchez@iciq.es (I.S.-M.); jrgalan@ICIQ.ES (J.R.G.-M.); 2 Institut de Recherches sur la Catalyse et l’Environnement de Lyon, UMR 5256, CNRS, Université Claude Bernard Lyon 1, 2 Avenue A. Einstein, 69626 Villeurbanne, France; 3 ICREA, Pg. Llu’ıs Companys 23, 08010 Barcelona, Spain

**Keywords:** formic acid oxidation reaction, electrochemistry, electrocatalysis, energy storage, catalyst benchmarking, fuel cells, CO adsorption, poisoning

## Abstract

Energy production and consumption without the use of fossil fuels are amongst the biggest challenges currently facing humankind and the scientific community. Huge efforts have been invested in creating technologies that enable closed carbon or carbon neutral fuel cycles, limiting CO_2_ emissions into the atmosphere. Formic acid/formate (FA) has attracted intense interest as a liquid fuel over the last half century, giving rise to a plethora of studies on catalysts for its efficient electrocatalytic oxidation for usage in fuel cells. However, new catalysts and catalytic systems are often difficult to compare because of the variability in conditions and catalyst parameters examined. In this review, we discuss the extensive literature on FA electrooxidation using platinum, palladium and non-platinum group metal-based catalysts, the conditions typically employed in formate electrooxidation and the main electrochemical parameters for the comparison of anodic electrocatalysts to be applied in a FA fuel cell. We focused on the electrocatalytic performance in terms of onset potential and peak current density obtained during cyclic voltammetry measurements and on catalyst stability. Moreover, we handpicked a list of the most relevant examples that can be used for benchmarking and referencing future developments in the field.

## 1. Introduction

The impelling need of curbing the energetic and environmental crisis we are facing nowadays calls for a common effort of scientists, industries and government institutions to find affordable solutions to mitigate emission of greenhouse gases such as CO_2_. Reducing CO_2_ emission necessitates the use of closed-loop or carbon-neutral fuel cycles. Fuel cells (FCs) are low-to-zero carbon emission electrochemical power-generating devices characterized by high thermodynamic efficiency, modularity and versatility [1]. For this reason, FCs are expected to play a key role in the near future as next-generation power sources for transportation, stationary and portable applications. The use of molecular hydrogen via proton exchange membrane fuel cells is considered to be the benchmark fuel cell technology due to the high power density, favorable weight-to-power ratio, low temperature and fast start-up [2,3,4]. However, despite the good reliability, the low volumetric energy density of hydrogen is one of the main issues still hindering the widespread use of this technology [5]. Hydrogen is a gas at ambient conditions and must be stored under high pressure or liquefied at low temperatures. As such, growing interest has been paid towards the direct formic acid/formate (FA) electrooxidiation reaction, which can be used as a low-carbon energy vector/hydrogen carrier for exploitation in carbon-neutral fuel cycles [6,7,8,9,10,11]. FA fuel cycles are carbon neutral because FA can be directly produced by electrochemical CO_2_ reduction driven by renewable energy sources. The stored chemical energy can then be directly converted into electrical power in an FA fuel cell. In comparison to hydrogen, FA is a liquid/solid fuel with higher volumetric energy density [12]. Moreover, FA is safer than hydrogen because it can be stored and transported as a salt or dissolved in water. Finally, FA fuel cells (FAFCs) have higher theoretical cell voltage (1.45 V using oxygen as oxidant, compared to 1.23 V vs. fuel cells based on H_2_) and lower crossover limitations than those based on other liquid fuels like methanol (1.23 V) and ethanol (1.14 V), theoretically resulting in higher power density [6,13].

Most direct formic acid or formate fuel cells employ Pd- and Pt-based catalysts as the anode and cathode, respectively, as reviewed by An et al. [13]. The performance of oxygen-driven fuel cells is often limited by the high overpotentials required for the oxygen reduction reaction and the poor oxygen solubility in the catholyte [14,15]. However, the fuel cell voltage of 1.45 V (calculated as cathode electrode potential minus anode electrode potential) can be enhanced to a large extent by using alternative oxidants with higher standard potentials, like those proposed in Table 1. Indeed, fuel cells using hydrogen peroxide as an oxidant have been widely studied [13]. Ce^4+^ (typically in the form of Ce(NH_4_)_2_(NO_3_)_6_), with a standard potential of 1.61 [16,17] or 1.72 V [15,18,19] vs. NHE, depending on the source, is also attracting increasing interest. Filkenstein et al. explored the use of other oxidants like Cr_2_O_7_^2−^, HClO, MnO^4−^ or S_2_O_8_^2−^, the latter being the strongest oxidant (2.123 V vs. NHE), and they reviewed the main issues concerning each of them [15,20]. It should also be noted that the expected cell voltage is also increased by employing a fuel cell configuration where the cathode and the anode operate in acid and alkaline media, respectively, as it is shown in Table 1. For instance, Li et al. [21,22] and Han et al. [23] employed this acid cathode/alkaline anode concept using H_2_O_2_ and Ce^4+^ as oxidants, respectively.

Formic acid/Formate electrooxidation involves deprotonation and two electron oxidation reactions with a CO_2_ formation in acid, and CO_3_^2−^ in base. This reaction has a standard potential (E^0^) with values in the literature ranging from −0.17 to −0.25 V vs. NHE [13,24,25,26,27,28].

HCOOH **→** CO_2_ + 2H^+^ + 2e^−^

HCOO^−^ + 3OH^−^ → CO_3_^2−^ + 2H_2_O + 2e^−^

The complexity of the FA electroxidation reaction (FAEOR) results in sluggish kinetics and hinders fuel cell power density. Indeed, despite being investigated since the early 1960s of the twentieth century [29,30], the lack of electrocatalysts with suitable catalytic activity and durability is still the main concern limiting the competitiveness of FAFC and hinders its large-scale use [6,13,31,32]. Similar to other types of low-temperature fuel cells, FAFC are still dependent on PGM catalysts, in particular Pd and Pt. These catalysts are generally characterized by a low onset potential (as low as 0.1 V vs. RHE) but are limited in current density/durability, especially due to a severe poisoning effect primarily due to CO species. Several experimental and theoretical studies, primarily with respect to Pt, Pd and Au, have been performed with the aim of elucidating the reaction mechanism as has been recently summarized comprehensively in a review by Fang and Chen [31]. It is commonly accepted that FAEOR can follow two reaction pathways: (I) A direct pathway involving the formation of CO_2_ without the involvement of CO or (II) an indirect pathway, which entails CO formation followed by CO oxidation to CO_2_. The latter should be avoided because CO can poison the catalysts, and can only be oxidated at higher potentials, thus limiting the catalytic activity [31]. However, there is still debate on the precise nature of the reactive intermediate involved in the reaction. Several species have been proposed, including formic acid adsorbed to the catalysts in the formate (deprotonated) or formic acid (protonated), as well as formate in its bidentate or monodentate configurations [31]. Due to this uncertainty, in this review, we will take into consideration catalysts for both formic acid and formate oxidation—hence referring to both formic acid and formate generally as FA without focusing on the mechanism or the precise intermediates involved. We will instead direct our attention to the analysis of catalyst performance under different conditions. 

The poisoning of FA electrocatalysts by CO or other intermediates has directed the effort of researchers towards improving catalytic activity through nanostructuring, or by combining catalysts based on Pt and Pd with other elements. As an alternative to Pt and Pd, other compounds have been explored with the aim of substituting these precious PGM elements by more abundant and cost-effective materials, which may present even better stability or resistance to poisoning. Furthermore, the variety of electrode types such as disks, single lattice planes, nanoparticles, nanoparticle composites and alloys, in addition to the variety of conditions used for electrolysis such as pH ranging from 0–14, FA concentration ranging from 0.1–4 mol/L, varying electrolyte/buffer types and concentrations, make meaningful comparison of electrocatalyst difficult. As such, in this review, a comprehensive examination of electrocatalysts for electrooxidation of formic acid/formate is provided with a focus on the catalytic performance on three electrodes half-cell configuration with the aim of benchmarking both the catalytic activity and the electrochemical conditions. Benchmarking is an essential procedure in catalysis to define common standards, enable reproducibility and speed up the advancement of the research field. This is especially important in catalysis due to the differences in the intrinsic properties of the materials/electrodes, in the testing procedures, in the instrumentation and in the different definitions adopted [33,34].

### Methods for Comparing Electrocatalysts for FA Electrooxidation

In an effort to compare the various catalysts reported in the literature in a systematic way, we have taken into account cyclic voltammetry as the analytical technique and we have selected different electrochemical parameters: the onset potential (*E_onset_*), the maximum current density obtained at peak conditions (*j_max_*) and the current density obtained during cyclic voltammetry at different potentials ranging from 1 to 1.6 V vs. RHE. All of these parameters have been obtained from the forward scan, unless otherwise stated. In this work, all current densities have been normalized by the *geometric area of the electrodes,* as the geometric surface area is obtained in a facile and consistent manner and is the most relevant parameter for fuel cell applications. In some cases of interest, the mass and electrochemically active surface area (ECSA) normalized current densities have also been discussed because these parameters are helpful to understand the activity on a more molecularly defined basis. If necessary, data have been estimated by using the experimental information reported in the articles. All the potentials are reported with respect to the reversible hydrogen electrode (RHE) to account for the thermodynamic effect of the pH in the reaction. All currents and potentials, when not specified in this review, have been collected manually by digitizing the plots with Plot Digitizer software, with an accuracy of ±0.1 mA cm^−2^ and ±0.1 V. Any other information that could significantly affect the previous parameters, like the pH, the presence of buffer or the scan rate employed in the cyclic voltammetry, have also been specified in each case. We believe these parameters are the most appropriate to characterize catalytic activity and predict actual fuel cell performance. Other parameters directly linked to the catalyst durability such as stability during repeated cyclic voltammetry and chronoamperometry should also be considered for fuel cell applications. 

The first section of this review is dedicated to Pt-based catalysts as the first and most intensely investigated materials. We start by describing general CV features, we then present a review of the different types of catalyst starting from the simplest disk electrode, followed by specific Pt lattice planes, Pt with different crystal structures, Pt nanoparticles dispersed in a conductive matrix and ending with Pt alloyed with other metals. In a parallel structure, the second section reviews Pd-based catalyst. The last section addresses catalysts based on elements other than Pt and Pd as an alternative with special attention addressed to those constituted of non-noble metals. Moreover, the new trends for FA electrooxidation catalysts are briefly mentioned. Finally, in the conclusions, a summary of the catalytic performance of the different materials is provided including electrochemical parameters such as the *E_onset_*, *j_max_* (normalized to the geometric surface area, the electrochemical surface area and the mass of the Pt or Pd on the electrode). We include the geometric surface area-normalized current to account for the interest in expected fuel cell performance as well as ECSA and mass normalized current to account for the interest in understanding and comparing the underlying activity of the electroactive materials. It is also important to be aware of the fact that the catalytic activity and mechanism of a catalytic material can change when going from the bulk to the nanoscale—necessitating evaluation of current normalized to geometric surface area, electrochemical surface area and mass for a full comparison of material characteristics. Lastly, we include proposed benchmark catalysts and electrochemical conditions for catalysts based on Pt, Pd and non-PGM materials with which future researchers can compare new electrocatalysts for formate oxidation.

## 2. Platinum for Formic Acid/Formate Electrooxidation

### 2.1. General Features of Formic Acid/Formate Electrooxidation on Pt

**Figure 1:**
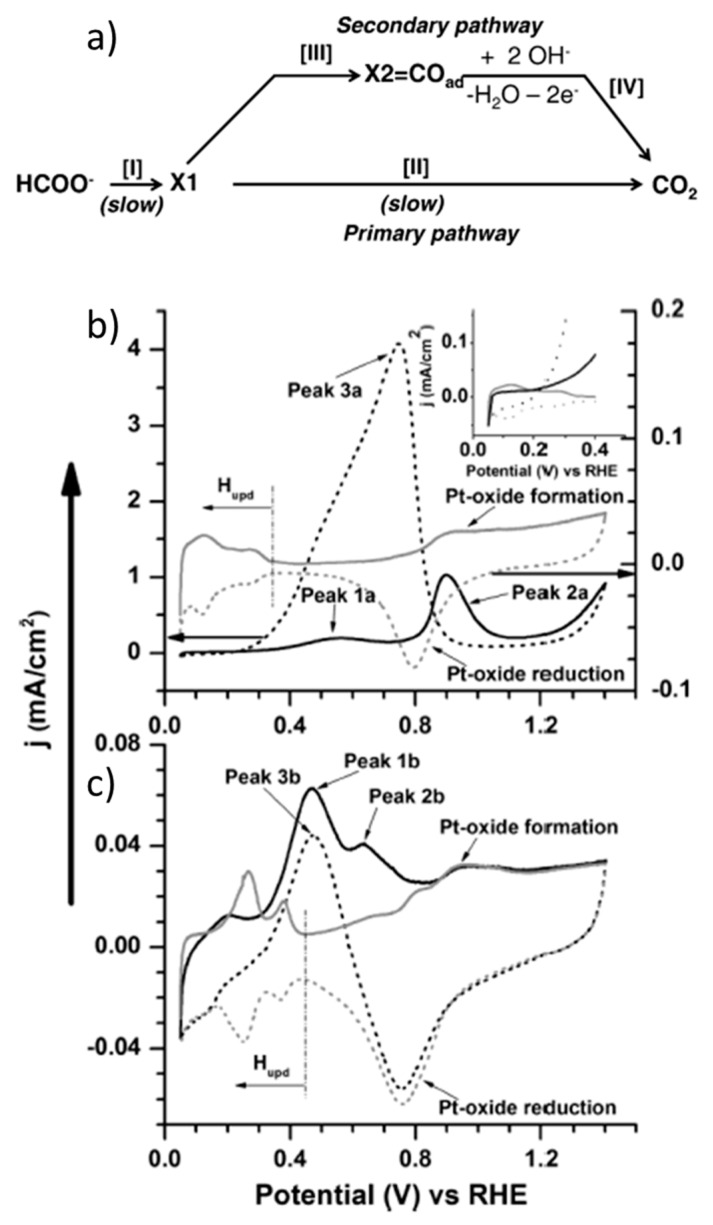
(**a**) Dual-pathway mechanism for HCOO^−^ oxidation proposed by Ref. [40] Cyclic voltammograms, at 20 mV s^−1^, of Pt disk in (**b**) 1 M HClO_4_ (grey); 0.2 M HCOOH + 1 M HClO_4_ (black), and (**c**) 1 M NaOH (grey); 0.2 M HCOONa + 1 M NaOH (black). Solid and dashed lines represent the forward and reverse scans, respectively. Reprinted with permission form Ref. [40].

Pt was the first metal catalyst employed in the FA electrooxidation reaction [29,35,36,37,38], before Pd [39] or any other metal [30], and it is one of the most widely studied catalysts to date. As mentioned above, it is generally accepted that formic acid/formate oxidation follows a dual-pathway mechanism composed of a main reaction pathway (known as direct or primary pathway) where formic acid/formate is oxidized to CO_2_ through a reactive intermediate and an indirect or secondary pathway involving an adsorbed poisoning species. For instance, John et al. proposed the dual-pathway shown in Figure 1a, where CO is the adsorbed intermediate in the indirect pathway, and a stable adsorbate with strong Pt−C bond, such as Pt-COOH, could be the reactive intermediate in the direct oxidation [40]. Figure 1b,c show cyclic voltammetry measurements with polycrystalline Pt disks as examples to describe the typical electrocatalytic behavior in acidic (0.2 M HCOOH + 1 M HClO_4_) and alkaline media (0.2 M HCOONa + 1 M NaOH), respectively. The CVs obtained with just electrolytes show two features: The under-potential hydrogen deposition (H_upd_) region, which typically takes place between 0 and 0.5 V vs. RHE at Pt, Rh, Pd and Ir electrodes [41], and the Pt oxide formation and reduction, which takes place at potentials above c.a. 0.7 V RHE in the forward (anodic) and reverse (cathodic) scans, respectively (Figure 1b). In the presence of HCOOH (black line in Figure 1b), two peaks are observed in the anodic scan. A first peak with an onset potential around 0.2 V vs. RHE derives from the direct HCOOH oxidation pathway, where the maximum current is limited by the concurrence of the adsorption of a poisoning intermediate (typically CO). Adsorbed CO is usually formed by the dehydration of formic acid in the H_upd_ region due to the reduction of adsorbed HCOOH or Pt-COOH by adsorbed hydrogen. A second oxidation peak takes place in the forward scan of the voltammetry at around 0.9 V vs. RHE, due to the oxidation of the adsorbed CO (indirect pathway) and subsequent formic acid direct oxidation, which is followed by oxidation of Pt to PtO at higher potentials. Then, upon decreasing the applied potential in the reverse scan, a third oxidation peak occurs along with Pt oxide reduction, due to the oxidation of more HCOOH molecules on CO-free Pt sites that have been cleared of adsorbed species (the direct oxidation pathway again).

HCOO^−^ oxidation in alkaline media (Figure 1c) follows a reaction mechanism very similar to HCOOH oxidation. However, the reaction kinetics are usually slower in basic media. As such, it is not surprising that FA oxidation studies on Pt are more common in acidic media (vide infra). In the case shown in Figure 1, the onset oxidation potential is very similar in both cases, around 0.2 V vs. RHE. The main advantage of using alkaline media is the improved tolerance to CO poisoning, as evidenced by the shift of the second oxidation peak in the forward scan towards lower potentials in Figure 1c, which is attributed to the presence of adsorbed OH [42,43]. The adsorption of OH, depending on the Pt face, may take place at potentials as low as 0.35 V vs. RHE, thus overlapping with the H_upd_ region [44,45]. Then, the comparison of acidic and basic media shows that, in the former case, the oxidation of adsorbed CO usually enhances the HCOOH direct oxidation, while in the latter case, CO is not always considered as the primary blocking species, as for example in the paper cited in this figure. In general, the higher the second peak during the forward scan and the higher the peak obtained in the reverse scan with respect to the first oxidation peak in the forward scan, the higher the contribution of the indirect oxidation pathway. More evidence of the CO poisoning effect is the suppression of the hydrogen desorption and adsorption peaks at low potentials.

Another important feature is that Pt oxide is known to be electrocatalytically active for formic acid oxidation in acidic media at potentials close to oxygen evolution reaction, while platinum catalysts usually present little to no activity after their oxidation in alkaline media [35,36]. This was confirmed by John et al. by differential electrochemical mass spectrometry (DEMS) [40]. Given the fast conversion of CO_2_ to CO_3_^2−^ in alkaline media, the authors employed an electrochemical cell where a Pt anode was sputtered on a porous Teflon membrane and mechanically supported on a porous stainless-steel frit interfaced to the vacuum system of the mass spectrometer, with which they monitored the CO_2_ generated during the CV. In this way, the authors observed that Pt activity in basic media was limited to the potential range of 0.2–0.7 V vs. RHE. Based on different potential step-linear sweep voltammetry and adsorbate stripping measurements, they stated that formate is not only the reactive intermediate in the direct oxidation pathway, but also the intermediate in CO formation in the H_upd_ region. Joo et al. [46], instead, claimed the absence of formate intermediate under similar reaction conditions, using in situ attenuated total reflection surface-enhanced infrared adsorption spectroscopy (ATR−SEIRAS), and they proposed a similar dual-pathway mechanism, but involving linearly adsorbed CO and bridge-bounded CO as a reactive intermediate and poisoning species, respectively. Other in situ spectroscopic techniques, like attenuated total reflection–Fourier transform infrared spectroscopy (ATR−FTIR) or electrochemical shell-isolated nanoparticle-enhanced Raman spectroscopy (EC−SHINERS), as well as density functional theory (DFT) calculations, have also been employed to study the formic acid/formate electrooxidation mechanism, as recently reviewed [31]. This mechanism is still matter of discussion, especially regarding the nature of the reactive intermediate for the direct oxidation pathway, the CO formation mechanism and even a hypothetical third indirect pathway through a different adsorbed intermediate (triple path). As an example, depending on the study, bridge-bounded formate is found to be a reactive intermediate [47,48], or a poisoning species [49,50]. However, this review is rather focused on the benchmarking catalysts and operation conditions. This section, in particular, will review some of the most interesting cases of Pt-catalyzed formic acid/formate oxidation, which are summarized in Table 2.

### 2.2. Monometallic Pt Catalysts

The simplest electrode configuration is the Pt disk electrode, which has been extensively employed for fundamental studies as many of those mentioned above. A flat surface of typically less than 0.1 cm^2^ geometric area limits the obtained current densities to less than 10 mA cm^−2^ [40,51,52,53,54,55,56,61]. Other basic Pt electrodes configurations are Pt net [57], Pt bead [58] and Pt single-crystalline electrodes, especially those containing the (111) exposed to the solution given the greater tolerance to CO poisoning of this specific lattice plane [59,60,81]. While Pt disk electrodes have the advantage of simplicity and ease of use, several authors employ Pt nanoparticles deposited on an electrode (typically glassy carbon disk) as reference materials to compare their further developed electrocatalysts [62,64,65,72]. These Pt nanoparticles usually show higher electrocatalytic activity than Pt disks under comparable conditions. For instance, Habibi et al. electrochemically deposited Pt nanoparticles on a ceramic carbon electrode and obtained a maximum current density of around 11 mA cm^−2^ at 0.8 V vs. RHE, with 0.5 M HCOOH + 0.1 M H_2_SO_4_ [62]. Yang et al. [52]. studied the improvement of Pt disk with a polyaniline (PANI) porous film (PANI/Pt). PANI provides a higher electrode area (53.69 cm^2^ vs. 1 cm^2^ bare Pt) and the ability to transfer protons via H^+^-doping or H^+^-dedoping, which could be very useful in view of the membrane electrode assemblies used in direct formic acid fuel cells. However, its stability has only be confirmed up to pH 5. PANI/Pt and Pt electrodes were tested with 0.1 M H_2_SO_4_ electrolyte and either 1 M HCOONa or 1 M HCOOH, and PANI/Pt showed lower poisoning by CO adsorption and higher current densities than Pt, especially in the formate solution, where the current densities of the direct HCOO^−^/HCOOH oxidation peak were 23 and 2.5 mA cm^−2^ with PANI/Pt and Pt, respectively. 

**Figure 2:**
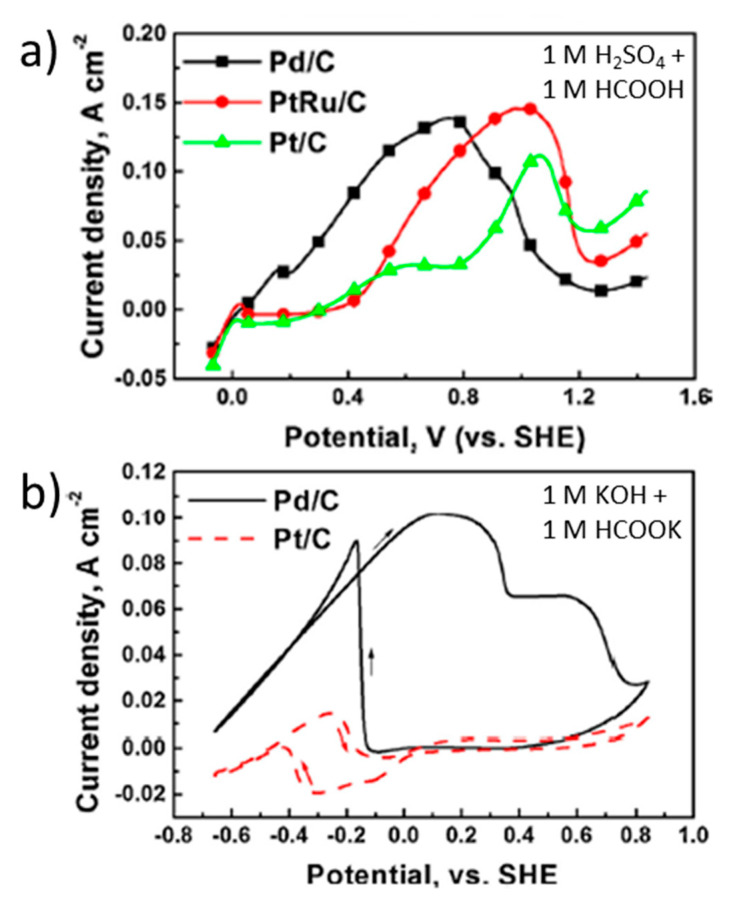
(**a**) Linear sweep voltammograms, at 20 mV s^−1^, of Pd/C, PtRu/C and Pt/C catalysts in 1 M HCOOH + 1 M H_2_SO_4_, (**b**) Cyclic voltammetry, at 20 mV s^−1^, of Pd/C and Pt/C catalysts in 1 M HCOOK + 1 M KOH. Reprinted with permission from Ref. [70].

Another way to decrease the Pt loading is to disperse the metal nanoparticles on a carbon matrix, which can be deposited in the form of an ink on an electrode support. Indeed, this is one of the most typical method to prepare the electrodes studied in direct formic acid or formate fuel cells [82,83]. The most common carbon matrix employed as support for Pt and other metal nanoparticles is carbon black (also known as Vulcan XC–72) [69,70,72]. For example, several authors have studied Pt/C anodes with 20 wt. % Pt obtaining peak current densities ranging from 4.4 to 13.4 mA cm^−2^ at a peak potential of around 1 V vs. RHE in acidic media [67,68,69]. Of course, the current density directly depends on the amount of active catalyst. Thus, Yu et al. obtained a peak current density of 110 mA cm^−2^, under similar conditions (pH around 0), with a Pt/C anode containing 40 wt. % Pt. These authors also compared the electrocatalytic activity for formic acid oxidation of this Pt/C catalyst with that of Pd/C and Pt_50_Ru_50_/C analogous anodes (40 wt. % metal and particle size of 2-4 nm in each catalyst) [70]. Figure 2a shows a cyclic voltammetry performed at 20 mV s^−1^ with 1 M HCOOH + 1 M H_2_SO_4_. Lower peak current density (110 mA cm^−2^ at 1.1 V vs. RHE) was obtained with Pt/C compared to Pd/C (140 mA cm^−2^ at 0.8 V vs. RHE) and PtRu/C (145 mA cm^−2^ at 1.0 V vs. RHE). Indeed, the latter value is the highest geometric area-normalized peak current density found in the literature for Pt-containing catalysts, either in acidic or in alkaline media. However, the onset potential for formic acid oxidation, which is another important parameter to define a proper catalyst for fuel cell application, is lower for Pd/C (around 0.1 V vs. RHE) than for Pt/C (0.2 V vs. RHE) and for PtRu/C (0.3 V vs. RHE). Moreover, based on Figure 2b, the authors claim that in alkaline media (1 M HCOOK + 1 M KOH), Pt/C does not provide any significant catalytic activity at potentials below 1.7 V vs. RHE, unlike Pd/C, which shows 102 mA cm^−2^ at 1 V vs. RHE. This is the base statement on which the authors address the development of a membraneless Direct Formate Fuel Cell using a Pd/C anode and a PtC cathode. Given the hypothetical inertness of the Pt cathode drawn from this figure, against a possible formate crossover, the use of any alkaline anion-exchange membrane would not be required, which was the final objective of this fuel cell design. However, this statement could be questioned because, under these conditions, Pt/C showed a non-negligible peak current density of ~15 mA cm^−2^ near 0.6 V vs. RHE (Figure 2b). Nevertheless, this paper demonstrates the better performance of Pt in acidic media vs. basic media and the superior electrocatalytic behavior of Pd compared to Pt, especially in the latter conditions, which can likely be attributed to the strong inhibiting effect of CO and OH adsorption on Pt electrodes, as discussed above.

A novel Atomic Layer Deposition (ALD) method to deposit Pt on carbon black previously attached to carbon paper was developed by Hsieh et al. [72]. They deposited the Pt from a MeCpPtMe_3_ precursor and high-purity oxygen on the carbon black, and they tested the catalyst at 1 M HCOOH + 1 M H_2_SO_4_. The reduction of the as-synthesized Pt oxide catalyst was carried out at different temperatures. They showed that the decrease in the reduction temperature from 450 to 150 °C increases the Pt electrocatalytic activity and hinders its deactivation by adsorbed poisoning species (CO). This was attributed to the beneficial influence of the Pt-O species generated by ALD, which provided a large number of active sites to oxidize CO, like Pt-O or Pt-(OH). Other carbon supports were explored by Habibi et al. [62], who modified carbon electrodes by depositing either carbon nanoparticles (CNP) or reduced graphene oxide (RGO) prior to the dispersion of Pt nanoparticles on this support and evaluated their electrocatalytic activity in formic acid oxidation in acidic media. Both catalysts showed very significant peak current densities, 27.7 and 38.9 mA cm^−2^ at 0.9 V vs. RHE with the platinum nanoparticles deposited on RGO- and CNP-based electrodes, respectively. Along with another catalyst based on PtBi alloy [69], this catalyst of Pt nanoparticles on CNP showed the lowest onset potential for formic acid oxidation found in the literature with Pt-based catalysts, around 0.1 V vs. RHE, which makes it competitive with Pd-based catalysts in acidic media (vide infra). Additionally, by optimizing the operation conditions with the Pt/CNP catalyst, the authors concluded that the maximum current density is reached with a formic acid concentration of 1 M, above which the catalyst performance is limited by saturation by adsorbed CO [62]. Different supports than carbon are also employed to deposit the Pt nanoparticles [62]. For instance, Pisarek et al. tested Pt and Pd nanoparticles (0.2 mg metal cm^−2^) deposited by magnetron sputtering on TiO_2_ nanotubes [73], and they obtained a similar maximum current density (around 14 mA cm^−2^) with the Pt/TiO_2_ catalyst when compared to Pd/TiO_2_ but at a significantly higher potential (1.0 and 0.4 V vs. RHE for Pt/TiO_2_ and Pd/TiO_2_, respectively) under the same operation conditions (0.5 M HCOOH + 0.5 M H_2_SO_4_).

### 2.3. Bimetallic and Trimetallic Pt-Based Catalysts

In order to decrease the loading of PGM on electrodes with improved performance, Liang et al. recently explored the use of Pt and Pd monolayers deposited on different single crystal electrodes (Au(111), Ir(111), Pd(111), Rh(111), Ru(0001) and Pt(111)), with 0.5 M HCOOH + 0.1 M HClO_4_ [74]. Among all of catalysts, the monolayers deposited on Au(111) showed the highest electrocatalytic activity, by far, in the forward scan of cyclic voltammetry. Moreover, in situ electrochemical infrared reflection absorption spectroscopy (IRRAS) found no evidence of adsorbed CO on Pt/Au(111), which was attributed to the ability of adsorbed Pt/Au(111) to form high coverage of OH, based on DFT calculations. This explains why the Pt/Au(111) surface has an outstanding performance as compared to other single crystal-supported catalysts and is much more active for formic acid oxidation and less poisoned by CO than Pt(111). Interestingly, this Pt/Au(111) strongly adsorbs CO, indeed, but its ability to adsorb OH is even more remarkable, and this advises that catalysts prone to CO poisoning should not be discarded, as long as they are able to oxidize the CO.

Other strategies to improve the electrocatalytic performance of Pt and other metal catalysts is the employ of metal alloys or the development of highly porous nanostructures. Besides the increase of Pt catalyst dispersion (i.e., decrease of Pt loading) on the electrode, the other main challenges when using these catalysts are the higher tolerance to adsorbates poisoning and improved electrode stability. In this sense, the alloy of Pt with other metals has been studied for many years and one of the most common alloys is PtRu. Apart from the previously cited paper by Yu et al. [70], there are several studies using PtRu alloys as bifunctional electrocatalysts for the oxidation of formic acid and other small molecules [76,77,84,85,86,87]. It is widely accepted that Ru sites nucleate oxygen-containing species more easily than pure Pt sites (at 0.2–0.3 V lower potential) [85] and, in organic molecules oxidation, Pt-Ru catalysts follow a bifunctional mechanism where CO is adsorbed on either Pt or Ru sites and further oxidized by these oxygen species nearby Ru atoms. This is the reason for the improved poisoning resistance of the PtRu alloy, with respect to Pt. For instance, Chen et al. [76] developed an electrocatalyst based on PtRu surface-alloy particles, with very low Pt loading, that obtained a higher maximum current density (2.2 mA cm^−2^ at 0.6 V vs. RHE) than the commercial PtRu catalyst (0.9 mA cm^−2^ at 0.6 V vs. RHE) for formic acid oxidation (0.1 M HCOOH and 0.1 M HClO), while a pure Ru catalyst showed negligible activity. However, the onset potentials reported in this article are surprisingly extremely low, around −0.1 V vs. RHE, calculated from the experimental data reported. 

**Figure 3:**
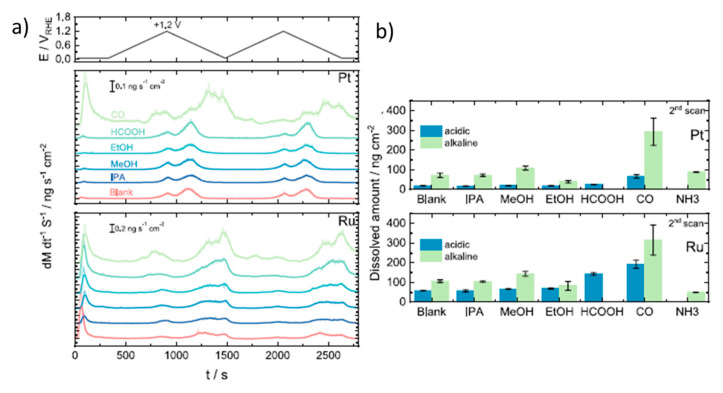
(**a**) Potential sweep profile along with dissolution rates of Pt (top) and Ru (bottom) for the PtRu 1:1 catalyst recorded during cyclic voltammetry from +0.05 to +1.2 V vs. RHE, at 2 mV s^−1^, in 0.1 M HClO_4_ and in the presence of various fuels, i.e., 0.1 M HClO_4_ + 0.05 M fuel (in the case of CO, the electrolyte is saturated). (**b**) Absolute dissolved amounts of Pt (top) and Ru (bottom) in the presence of each fuel both in 0.1 M HClO_4_ and 0.05 M KOH solutions. Reprinted with permission from Ref. [77] Further permissions related to the material excerpted should be directed to the ACS.

Kormányos et al. studied the electrocatalytic activity of PtRu for the oxidation of formic acid and other fuels under both acidic and alkaline conditions [77]. With an onset potential of 0.2 V vs. RHE and a peak current density of 5.1 mA, formic acid oxidation presents better electrocatalytic performance than methanol and ethanol. To understand the electrochemical mechanism more intimately and, in particular, catalyst deactivation, the authors tracked the metal dissolution events that occurred under the potential sweep in situ by ICP-MS [88,89]. For instance, Figure 3a shows the real-time Pt and Ru dissolution rates during two potential scans at 2 mV s^−1^ from +0.05 V to +1.2 V vs. RHE with 0.1 M HClO_4_ and 0.05 M of each fuel, including the blank (with only 0.1 M HClO_4_). In the blank experiments and with all fuels (except CO), Pt dissolution starts during the forward scan at potentials between 0.91 and 0.95 V vs. RHE, and the onset potential for Ru dissolution is lower, between 0.82 and 0.87 V vs. RHE. Interestingly, there is a cathodic dissolution of Pt and Ru that is even more pronounced than the anodic one. The cathodic dissolution of Pt is due to the reduction of the previously formed PtO_x_ layer, while in the case of Ru, it is attributed to the reduction of RuO_2_·2H_2_O and/or Ru(OH)_3_. Moreover, an additional anodic Ru dissolution peak was found when the authors extended the upper limit potential to +1.5 V, which was attributed to the formation of RuO_4_. From the quantitative results shown in Figure 3b, the dissolution of Ru is much stronger than that of Pt in acidic media. The authors also did similar experiments in alkaline media. Although the authors did not test the formate electrooxidation under these conditions, it can be observed that both Pt and Ru dissolution increase at higher pH, although this increase is much more pronounced in the Pt (14-fold increase) than in the Ru (4-fold increase). The Ru dissolution from this PtRu catalyst in alkaline media was much lower than that reported for pure Ru [88], which was explained as a stabilization effect of Pt on Ru. Besides, in the presence of formic acid, with respect to other fuels, the dissolution rates were found to be higher. The greatest dissolution features were observed when the experiments were performed in a CO-saturated electrolyte implying that the indirect oxidation pathway (i.e., through CO adsorption and oxidation) plays a key role in formate electrooxidation using PtRu as the catalyst.

**Figure 4:**
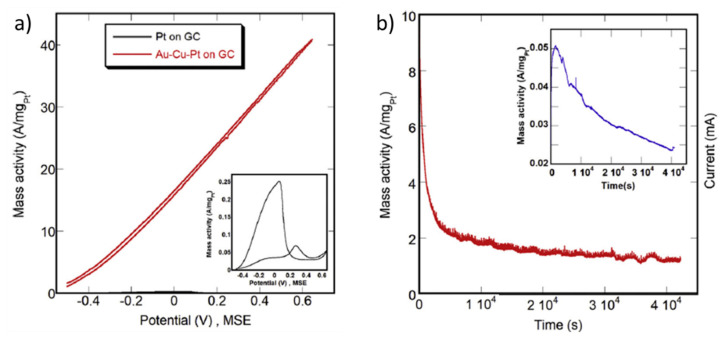
(**a**) Cyclic voltammetry, at 50 mV s^−1^, and (**b**) chronoamperometry at 0.7 V vs. RHE, of PtAuCu (2.6 at. % Pt) in 2 M HCOOH + 0.1 M HClO_4_. Inset figures show the measurements performed on bare Pt. Reprinted with permission from Ref. [80].

Other Pt-based alloys have been tested in formic acid/formate electrooxidation such as PtFe [79], PtAu [67,90], PtAg [65], PtCo [66], PtCu [68], PdPt [39,71,73,91,92], PtBi [69,93] or PtSn [68,93,94] (Table 2). Indeed, the use of Bi, Sb, As, Pb, Sn and Ge as dopants for Pt has been investigated from several decades ago, finding optimum loadings of around 50% in most cases [93,94]. Adzic et al. even added these dopants in situ, by electrodeposition on Pt electrodes, leading to enhanced electrooxidation of formic acid. By cyclic voltammetry, at 50 mV s^−1^, in 0.265 M HCOOH + 1 M HClO_4_, maximum current densities of 7.2, 20.7, 46.0 and 70.2 mA cm^−2^ were obtained in the presence of Cd^2+^, Ti^+^, Bi^3+^ and Pb^2+^, respectively [95]. In this work, the authors attributed the superior performance of the catalysts, with respect to pure Pt, to the suppression of the hydrogen adsorption on Pt and the subsequent hindering of CO adsorption. In a work from the present year, Menshikov et al. compared the electrocatalytic performance of a Pt/C catalyst with that of PtCu/C and Pt/SnO_2_/C catalysts in acidic media [68]. They found an onset potential of 0.2–0.3 V vs. RHE in all cases, but much higher peak current density on PtCu/C (30.8 mA cm^−2^) and Pt/SnO_2_/C (32.8 mA cm^−2^) with respect to Pt/C (30.8 mA cm^−2^). On the other hand, in the chronoamperometry measurements, the stability of these catalysts strongly depended on the applied potential. Among the three catalysts, PtCu/C is the most stable after 30 min at 0.87 V vs. RHE, while Pt/SnO_2_/C was the most stable at lower potentials, i.e., 0.6 V vs. RHE. In another recent work, PtBi nanoplates were deposited on carbon black and tested for formic acid and glycerol oxidation showing superior electrocatalytic performance in both reactions with respect to a Pt/C catalyst [69]. In the case of formic acid oxidation, the onset potential was decreased by 220 mV and the peak current density was increased from 4.4 (Pt/C) to 46.1 mA cm^−2^ (PtBi/C), calculated from the mass activity. The suppression of poisoning by CO on PtBi/C was confirmed by in situ FT–IR, which could explain the superior performance of PtBi/C in comparison to Pt/C. This promoting effect of Bi on Pt for the selective oxidation of organic molecules is commonly attributed either to the blocking by Bi of specific sites for CO adsorption or to the enhanced adsorption of OH species in the vicinity of Bi as confirmed by FTIR [96,97,98]. In the mentioned study, Wang et al. claimed that the mass activity that they obtained with the PtBi/C catalyst, 9.06 A mg/Pt, was the highest electrocatalytic activity ever reported [69]. However, in 2019, Xie et al. reported a catalyst based on a trimetallic PtAuCu alloy catalyst, which was subjected to a dealloying process to selectively remove Cu from the surface and showed a maximum mass activity of 54.8 A mg/Pt, with only 1.3 at. % Pt [80]. The results obtained in this work have not been added in Table 2 because the experimental data to convert the mass-normalized current density into geometric area-normalized current density is not clear. Besides, the most groundbreaking result obtained by Xie et al. is the electrocatalytic performance of a similar catalyst, based on Au-Cu-Pt, but with 2.6 at. % Pt, which has been included in a summary table in the conclusions section, with the best-performing catalysts. In this case, the mass activity is lower than in the previous case but, surprisingly, the catalyst did not reflect any sign of CO–poisoning or passivation at most positive potentials (see Figure 4a). This is a unique case among the Pt-based catalysts. The current density continuously increased with the potential, at least, up to 1.3 V vs. RHE, and showed a very good stability (Figure 4b). The authors attributed this outstanding performance to the specific surface electronic structure of the Au–Pt dominated alloy, the high level of Pt dispersity and a synergistic effect of Au, Cu and Pt in the formic acid oxidation reaction.

In the case of PtPd catalysts, they will be mentioned in the next section. Another catalyst with outstanding electrocatalytic performance for formic acid oxidation is the PtCo alloy supported on the polyaniline/SBA-15 nanocomposite developed by Laskenari et al. [66]. First, the PANI/SBA-15 nanocomposites were synthesized through in situ emulsion polymerization. Then, Co and Pt (in that order) were electrodeposited on the surface. Cyclic voltammetry was performed, in 0.5 M H_2_SO_4_ + 0.5 M HCOOH, with PtCo/PANI/SBA-15 catalysts synthesized from different concentrations of the Co precursor solution (Co(NO_3_)_2_), the Co-free catalyst and a reference PtCo. All the catalysts have similar onset potentials (between 0.3 and 0.4 V vs. RHE). However, important differences were found in terms of current density values. The optimum PtCo/PANI/SBA–15 obtained a peak current density of 63.8 mA cm^−2^, which outperformed that of Pt/PANI/SBA–15 (33.2 mA cm^−2^) and PtCo (19.5 mA cm^−2^). However, an exponential decay of the catalytic activity was found with time. As in most cases using Pt-based catalysts, this weakness is mainly attributed to the ease of Pt to be poisoned by CO.

**Figure 5:**
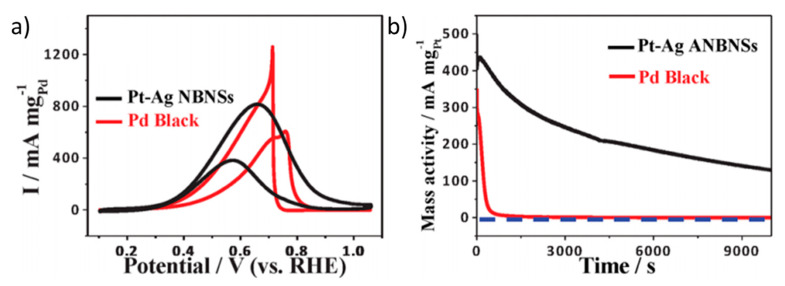
(**a**) Cyclic voltammetry, at 50 mV s^−1^, of Pt-Ag nanoballoon nanoassemblies and Pd black, and (**b**) chronoamperometry at 0.56 V vs. RHE on the two catalysts, in 1 M HCOOK + 1 M KOH. Reprinted with permission from Ref. [65].

Han et al. developed a catalyst based on a PtAg alloy with a special nanostructure that they called nanoballoon nanoassemblies (NBNS) [65]. It consisted of the synthesis of Ag templated with flower-like 3D-multibranched morphology, where SPA is used as a surfactant to prevent Ag agglomeration and the subsequent galvanic replacement reaction with K_2_PtCl_4_ is used to deposit Pt atoms on the surface. In this case, the catalyst was tested in alkaline conditions, and it showed a slightly better onset potential (0.2 V vs. RHE) and a much higher peak current density (32.6 mA cm^−2^) than a Pt black catalyst (0.3 V vs. RHE and 32.6 mA cm^−2^, respectively). Based on these results, PtAg/NBNS is even more active than Pd black (Figure 5a). The authors attribute this enhancement to a higher resistance of PtAg/NBNS to CO poisoning, as deduced from the relative peak intensities observed in the cyclic voltammetry. This would explain the different result obtained by chronoamperometry measurements (Figure 5a), where PtAG/NBNS retained 36% of its initial activity after 2.8 h, while Pd black totally deactivates after a few seconds. However, this stability should be still improved for application in fuel cells.

Regarding optimum cell operation conditions, several conclusions could be also drawn from the reviewed literature on Pt catalysts. From Table 2, one can determine that most of the studies on Pt-based electrodes are focused on acidic conditions. This is due to the faster kinetics of this metal under these conditions, with respect to alkaline media, and the avoidance of OH poisoning effect. On the other hand, the main hinderance derived from this metal, the ease of CO poisoning, could be overcome by using certain supports or metal alloys as those reported herein. Besides, it should be noted that most of studies have been carried out under extreme pH conditions (i.e., pH closed to 0 or 14). However, several researchers show a volcano-type behavior of the electrocatalytic activity with the pH. Optimum pH conditions are usually found at around FA pKa (i.e., 3.75) [46,55,58,60,99,100], although Haan et al. did not reach the maximum electrocatalytic activity in the pH range 0–5 [101], and Ferre–Vilaplana et al. obtained an optimum pH of 5.5 [81]. In any case, from the literature review, the convenience of operating at pH closer to neutral conditions rather than strongly acidic or basic conditions seems clear. The use of a buffer is strongly recommended to maintain intermediate pH values on the electrode surface without any effect of the produced protons on the molar ratio HCOOH/HCOO^−^. One could expect some interference from the adsorption of anions like phosphate, for example, in the case of the Na_2_HPO_4_/NaH_2_PO_4_ buffer. Surprisingly, some studies in the literature neglected the coverage of these anions in their calculations, due to a similar pH-dependence of HCOOH/HCOO^−^ oxidation found in different buffered solutions (Na_2_HPO_4_/NaH_2_PO_4_ vs. HClO_4_/ClO_4_^−^ vs. HSO_4_^−^/SO_4_^2−^) [46,101]. However, buffer solutions must be employed carefully, as reported by Abdelrahman et al. [60] and Perales-Rondón et al. [55], who did find a detrimental effect of phosphates in the formic acid oxidation reaction. Phosphate anions can be strongly adsorbed on the surface, blocking reactive surface sites and thus decreasing the obtained current densities.

Regarding the optimum formic acid/formate concentration, several works found a linear dependence of the obtained current densities with the studied fuel concentration range up to 0.2 M HCOONa [40] or 0.5 M HCOOH [66], while Okamoto et al. [57] and Habibi et al. [62] found surface saturation at HCOOH concentrations above 1 M and Yang et al. [52] found maximum current densities in the range of 0.8–2 M HCOONa. Thus, in general terms, it seems that the optimum formic acid/formate concentration is of the order of 1 M, regardless of the electrolyte. Indeed, most direct formate fuel cells reported in the literature are fed by 1 M HCOOK or 1 M HCOONa [13], although direct formic acid fuel cells are reported to employ HCOOH concentrations ranging from 0.5 to 10 M [28,102]. Finally, studies performed in a three-electrode configuration exploring cell temperatures higher than room temperature are scarce [57,61]. The higher the temperature, the faster the formic acid/formate oxidation kinetics. However, in fuel cell applications, regardless of energy consumption considerations, this parameter is limited by membrane requirements (temperatures typically not higher than 100 °C). Then, we think that higher efforts should definitely be conducted in operating three-electrode configuration cells at conditions closer to those typically employed in direct formic acid/formate fuel cell studies, i.e., temperatures up to 60–80 °C [13,70], at which the stability of most of the catalysts is yet to be checked.

### 2.4. Benchmarking Pt Formic Acid/Formate Electrooxidation Catalysts

According to this literature review, we propose five Pt benchmarking catalysts (see summary table in conclusions section): (i) The classical Pt disk, (ii) Pt(20 wt. %)/C, (iii) the PtBi/C catalyst with the minimum possible onset potential, 0.1 V vs. RHE [69] and, regarding peak current density, we highlight (iv) the PtRu/C [70] catalyst and (v) the PtAuCu dealloyed catalyst [80], which showed the highest reported geometric area- and mass-normalized activities, respectively. In the latter case, moreover, the catalyst has an outstanding stability and tolerance to CO poisoning.

## 3. Palladium for Formic Acid/Formate Electrooxidation

### 3.1. General Features on Formic Acid/Formate Electrooxidation on Pd

Palladium is a transition metal with high reactivity exhibiting good catalytic activity with respect to many reactions ranging from cross coupling reactions [103] to Sonagashira and Heck reactions [104]. Additionally, palladium is electrically conducive and electrocatalytically active towards a variety of reactions including hydrogen evolution [105,106], oxygen reduction [107] and formate oxidation [32,108]. In the case of formate oxidation, palladium and its alloys exhibit an onset potential ranging from 100 to 300 mV vs. RHE and have a maximum current density of 2.8–230 mA/cm^2^. Moreover, although Pd is less prone to being deactivated by CO as compared to Pt [13], Pd still tends to deactivate at high applied potentials due to the accumulation of adsorbed intermediates that poison the catalyst surface and deactivation due to the formation of palladium oxide as discussed further elsewhere [32,108]. 

There is ongoing debate as to the precise nature of the adsorbed intermediates that lead to catalyst deactivation. Spectroscopic studies of the surface of Pd anodes during FA oxidation have established that CO is not likely the primary poison at high potentials [109], rather it has been suggested that CO is formed via the reduction of CO_2_ at an open circuit and not as an intermediate of the indirect oxidation pathway [108]. Nonetheless, the amount of poisoning by adsorbed intermediates is greatly affected by the precise surface structure of the material, and several groups have investigated the effect of the exposed lattice plane on the electrochemical formate oxidation activity. 

Similar to Pt, attempts to increase the catalytic activity of FAEOR while decreasing palladium loading have been made primarily through nanostructuring of the catalyst and through alloying palladium with other metals such as copper, gold, indium and silver. In this section, we review catalysts based on palladium, ranging from planar polycrystalline palladium surfaces, single crystal lattice planes, palladium monolayers, palladium nanoparticles and alloys based on palladium. A selection of relevant references is summarized in Table 3, and some exemplary studies are presented more in depth in the following section. Finally, benchmark conditions and systems are proposed based on ease of use, catalyst performance, and prevalence in the literature.

### 3.2. Monometallic Pd Catalysts

**Figure 6:**
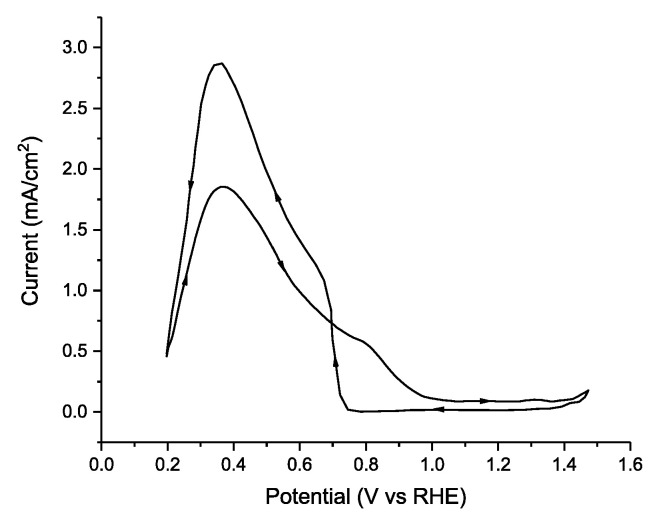
CVs of a planar palladium disk electrode in a solution containing 1.0 M HCOOH + 0.5 M H_2_SO_4_ at a scan rate of 70 mV s^−1^. Digitized from Ref. [30].

The simplest electrode based on palladium is an analytically pure, polycrystalline, palladium disk electrode. The first reports using Pd disk electrodes date back to 1965 [131], and the first comprehensive comparisons of the noble metal series occurred in the classic 1973 paper from Capon and Parsons [30]. In that paper, custom-made Pd disk electrodes were fabricated, and cyclic voltammetry was conducted with a 1.0 M solution of HCOOH in 0.5 M H_2_SO_4_, yielding a maximum current density of 2.8 mA cm^−2^ on the reverse scan (Figure 6). In this case, deactivation of the catalyst at high potentials is readily apparent in that the peak current in the anodic scan is reached at a potential of +0.4 V vs. RHE and the current declines significantly and a mass transport limited regime is not observed—which is typical of Pd electrodes in FAEOR. Further studies by Capon and Parsons demonstrated a strong dependence of the catalytic current on pretreatment of the Pd electrode and they present evidence against Pd deactivation via the formation of PdO, favoring poisoning of the electrode surface via adsorbed intermediates [132], which was corroborated by on-line mass spectrometry experiments by Solis et al. [110]. While a polycrystalline Pd disk or Pd foil are the simplest electrodes to fabricate, the electrocatalytic electrochemical mechanism is likely to be dependent upon the surface atomic structure—especially the exposed lattice planes. 

**Figure 7:**
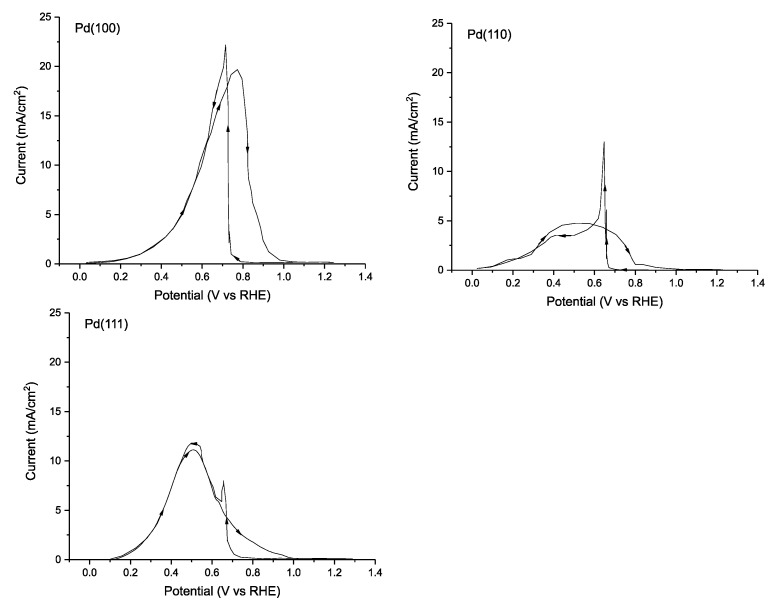
CVs of Pd single crystal electrodes with the (100), (111) and (110) lattice planes exposed of solutions containing 0.1 M HCOOH + 0.1 M HClO_4_ (pH ≈ 1.0) 20 mV s^−1^. Digitized from Ref. [111].

By comparing the electrocatalytic activity of the various lattice planes of Pd, one is able to evaluate the binding energies of FA and the adsorbed intermediates and develop a more molecularly defined mechanistic model. Hoshi et al. published results examining the electrocatalytic activity of single crystal Pd electrodes consisting of surfaces that expose the Pd(100), (111) and (110), lattice planes using 0.1 M HClO_4_ as electrolyte [111]. It was discovered that in these conditions, FA oxidation performance follows the order: Pd(100) > Pd(111) > Pd(110) with a maximum current density of 19.7, 11.0 and 4.8 mA cm^−2^ on the forward scan (Figure 7). This drastic difference in performance can be attributed to the relative binding energy and mode of the FA substrate, which affects both the reaction mechanism and the energetics of the intermediates. As is common with Pd, the single lattice plane catalysts are deactivated at high potentials. 

While the studies of Hoshi et al. [111] have provided insight to the relative activity of Pd towards FAEOR in the bulk, more recent studies by Choi et al. have examined the effect of exposed facets and defects in the nanostructuring of Pd FA electrooxidation catalysts [112]. Nanocrystals of different shapes have different lattice planes exposed—for example a cube has only {100} planes exposed, an octahedron only has {111} planes and an icosahedron contains both {111} and {211} planes [112]. Studies controlling the size and shape of Pd nanoparticles demonstrated that Pd nanoparticles do indeed exhibit shape-dependent catalysis [112]. For example, features such as the anodic peak potential and peak current are affected by the specific lattice planes exposed to solution and are in agreement with the bulk single crystal experiments in that the {100} lattice planes are more active than the {111} planes (Table 3) [111]. DFT of the FAEOR reaction intermediates and, more specifically, the binding energy of the intermediates provides insight and rationalization regarding the relative rate of formic acid/formate oxidation, the production of CO and catalyst poisoning on the specific lattice plane exposed [112]. For example, the Pd {100} surface binds HCOO− (i.e., formate bound through the O atoms rather than through C, −COOH) stronger than the Pd {211} and {111} by 220 meV and 110 meV, respectively. In contrast, the binding of COOH is relatively isoenergetic for each surface and helps explain why the icosohedra outperform both the cubes and octahedrons because –COOH is a precursor to CO poison intermediates [112]. While single crystal electrodes and shape-specific nanoparticles can provide mechanistic insight, they are relatively difficult to fabricate when compared to polycrystalline phases of Pd such as Pd black. 

**Figure 8:**
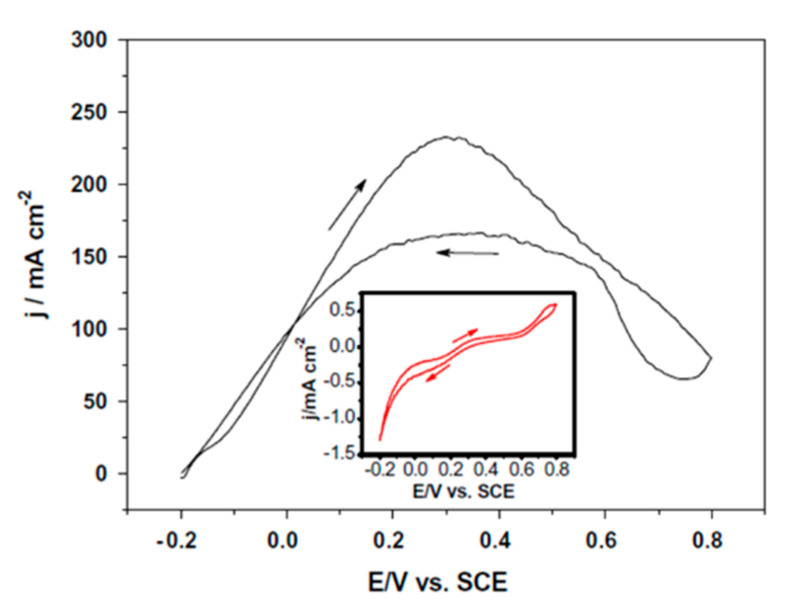
Cyclic voltammetry, at 10 mV s^−1^, of Pd nanoporous electrode, in 0.5 M HCOOH + 0.5 M H_2_SO_4_. The inset shows the CV of a Pd foil electrode under the same conditions. Reprinted with permission from Ref. [114].

Studies using pure palladium have examined Pd black in drop-cast inks for FA oxidation [71,113]. Pd black is essentially a polycrystalline or amorphous phase of Pd that is generally more catalytically active than bulk Pd due to its higher surface area and number of defect sites. The activity of Pd black is highly dependent on pH with an onset potential ranging from 0.1 to 0.8 V vs. RHE in acidic and alkaline conditions, respectively [71,113]. Furthermore, a higher peak current was achieved in the alkaline conditions of 27.3 mA cm^−2^ compared with 5.4 mA cm^−2^ in acidic conditions, albeit at a much higher over potential. This drastic difference in both maximum current density and current/potential dynamics suggests a change in electrocatalytic mechanism—possibly related to the protonation state of the formate/formic acid adsorbate and adsorbed intermediates, a factor that must be considered when comparing amongst catalytic systems. Additional studies using nanoporous Pd inks had onset potentials lower than 0.1 V vs. RHE and current densities up to 232 mA cm^−2^ in acidic conditions (at around 0.6 V vs. RHE), as shown in Figure 8 [114]. The high current density of this catalyst, the highest geometric area-normalized current density ever reported for formic acid oxidation, is likely due to the highly porous nature of the Pd synthesized by dealloying of an AlPd nanoribbon alloy. Indeed, this catalyst presents an outstanding electrochemically active surface area of 23 m^2^ g^−1^. While a comparison of catalysts with drastically different surface areas in a fair and consistent manner can be challenging, an increase in the current density by a factor of 40 for the nanoporous Pd over Pd black is remarkable. While Pd has been used as a pure material in inks, its loading can be decreased, and its amount thus reduced (thereby decreasing costs).

Similar to the strategies followed with Pt–based catalysts, the primary way that Pd loading is decreased is by mixing it with an electrically conducting substrate such as Vulcan carbon (carbon black) [53,70,115,116,117,118,119,120,121], graphene [121], carbon nanotubes [121], reduced graphene oxide [117,122] and TiO_2_ nanotubes [73]. The results of those studies show a strong correlation between catalyst loading, the support material and the precise solution conditions with maximum current densities ranging from 0.6 mA cm^−2^ to 140 mA cm^−2^. The latter value was obtained with a Pd/C anode containing 40 wt. % Pd for formic acid oxidation under acidic conditions, as previously observed in Figure 2a. In the case of examining the support material, an increase of catalytic current density was observed when comparing Vulcan carbon (3.6 mA cm^−2^), graphene (7.7 mA cm^−2^) and N-doped graphene/Carbon nanotubes (17.6 mA cm^−2^) under otherwise identical conditions [121]. In further support of this is a comparison that shows increased current when comparing a carbon support (40 mA cm^−2^) with a support based on reduced graphene oxide (57 mA cm^−2^) [117]. While the precise reason for the change in the catalytic activity when varying the support material is not precisely known, it is likely caused by variances in the metal dispersion, the electrical conductivity of the support, the electrochemical coupling of the support and catalyst, the electrochemically active surface area or other secondary steric effects that change the binding energy of the intermediates. While the catalyst loading of Pd can be decreased while maintaining catalytic current, as already shown, all catalysts based on Pd suffer from poisoning at high applied potentials and the maximum current and thus the maximum power density of FA fuel cells are limited by this poisoning. It is therefore of great interest to decrease or eliminate the Pd catalyst poisoning phenomenon. 

### 3.3. Bimetallic and Trimetallic Pd-Based Catalysts

**Figure 9:**
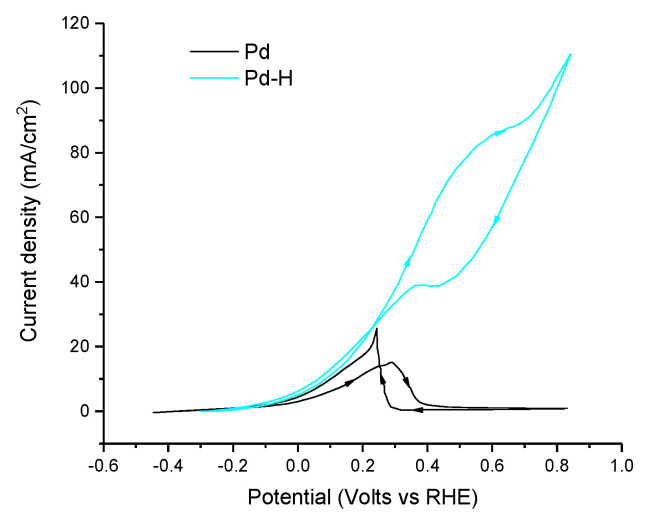
Comparison of Pd (black) with Pd-H (cyan) for formate oxidation in alkaline conditions, showing a significantly reduced poisoning effect for the initial Pd vs. Pd–H. Digitized from Ref. [123].

In order to decrease the observed poisoning of FA electrooxidation catalysts based on Pd, many authors have examined a variety of techniques, ranging from Pd–H [123], Pd monolayers on other metals [74], nanoparticle alloys [71,73,118,120,122,124,125,126,133,134], core shells [126], nanotubular alloys [127] and aerogel alloys [115,128,129]. Interestingly, it was shown that the poisoning effects for Pd-H was greatly reduced when compared with native Pd and the authors attribute this change in reactivity to hydrogen-mediated reduction of adsorbed CO to formaldehyde, which is then oxidized to carbonate (Figure 9) [123]. One important caveat to note in that study is that the Faradaic efficiency was not determined, and it cannot be ruled out that the rise in catalytic current is simply due to hydrogen oxidation and not formate oxidation as claimed—highlighting the importance of determining the Faradaic efficiency in electrocatalysis [123]. Another interesting case study examined the effect of the metallic underlayer when depositing Pd monolayers onto Ir(111), Pt(111) and Au (111) [74]. Under acidic conditions, the electrochemical performance of the Pd monolayer was 4.3 mA cm^−2^ on Ir, 15 mA/cm^2^ on Au and 55 mA cm^−2^ on Pt, in comparison to Pd(111), which had a maximum current density of 2.9 mA cm^−2^ in those conditions [74]. DFT on the reaction intermediates of the Pd monolayers shows a strong correlation between the electrocatalytic activity and the energetics of the adsorbed intermediates such as CO and other relevant species such as OH. For example, the best-performing Pd on Pt (111) had the highest binding energy of the –COOH intermediate and the lowest binding energy of the HCOO- intermediate [74]. In principle, the binding energy of adsorbed species can be controlled by factors such as, for example, precise energy levels, symmetry or atomic spacing, all of which can be tuned through the synthesis of alloys of Pd. 

There is a plethora of publications using alloys based on Pd, the full scope of which is beyond this review [71,73,115,118,120,122,124,125,126,128,129,133,134]. However, in a few exemplary studies, the maximum current, onset potential, propensity towards poisoning and stability can be improved when alloying Pd with metals such as Bi [124], Sn [135], Cd [124], In [125], Ag [71,115,118,126,127,128], Cu [32,128,129,134], Ce [118], Co [118,133], Ni [118,120,126], Pt [39,71,73,91,92] and Au [129] (Table 3). For example, when comparing Pd black with PdCu nanoparticles, the onset potential is decreased from 0.8 to 0.5 V vs. RHE and the maximum current is increased from 27.3 to 84.6 mA cm^−2^ under identical, alkaline conditions [71]. Another alloy based on PdCu consists of an aerogel synthesized using a sol-gel method and supercritical drying, which achieved a maximum current density 174 mA cm^−2^ [129]. It is noteworthy that in the previously mentioned work with Pd and PdCu nanoparticles, Chen et al. also explored the use of PdCuPt trimetallic alloy and this catalyst has a similar onset potential as PdCu, 0.5 V vs. RHE, but the highest current density among the three catalysts, 102.4 mA cm^−2^ at 1.2 V vs. RHE. Indeed, PdPt alloys have been studied for formic acid oxidation since 1966 [39], and typically show higher current densities than the pure metals, at least in the short term [91,92]. Even a small addition of Pt to Pd can be profitable. For example, in the work of Pisarek et al. [73], the best electrocatalytic performance was obtained with a Pd/TiO_2_ (0.1 mg Pd cm^−2^) containing a small amount of Pt (only 0.02 mg Pt cm^−2^), which showed an onset potential of around 0.1 V vs. RHE and a peak current of around 20 mA cm^−2^ at 0.3 V vs. RHE. This electrocatalytic performance overcame that of Pd/TiO_2_ and Pt/TiO_2_ catalysts (0.2 mg metal cm^−2^), both showing around 14 mA cm^−2^ of peak current density, as described in the previous section.

Abundant literature can be also found about Pd-based FA electrooxidation catalysts and catalyst systems with differences in Pd particle size, particle shape, electrode support, electrolyte and formate concentration, among others. All of these factors affect the observed electrocatalytic performance with maximum current densities over two orders of magnitude (from 2.8 to 232 mA cm^−2^). Given this enormous range in observed electrocatalytic activity, and the ongoing interest in formic acid/formate electrooxidation, it is critically important to establish reliable benchmarking protocols for the reliable comparison of catalysts [33]. Furthermore, as part of the best practices for electrocatalytic benchmarking, future work should normalize the observed current to the electrochemically active surface area (ECSA) and the mass of catalyst [33].

### 3.4. Benchmarking Pd Formic Acid/Formate Electrooxidation Catalysts

Table 5 in the Conclusions section presents a set of viable, working benchmark conditions including the precise nature of the Pd catalyst, the catalyst loading, the pH, electrolyte and formic acid/formate concentration as well as the expected parameter outputs such as approximate onset potential and maximum current density (normalized to geometric surface area, ECSA and mass of catalysts). The proposed benchmark systems for electrocatalytic oxidation of FA using Pd-based catalysts consist of (i) a Pd-disk electrode because of its ease of use and therefore reproducibility [30,110]; (ii) 40% Pd on Vulcan carbon because of its common use and high degree of accessibility [70]; (iii) PdNi on Ketjen carbon because it is one of the best-performing Pd-based alloys in terms of maximum current density (normalized by both geometric area and mass) [120]; finally, (iv) a Pd nanoporous electrode with the maximum geometric area–normalized current density [114]. Future publications should select one of these or a similar electrocatalytic system with which to compare new catalysts based on Pd for reliable analysis and comparison. 

In summary of the catalysts based on Pd for electrocatalytic formic acid/formate oxidation, they show remarkable electrocatalytic activity with onset potential as low as 100 mV vs. RHE and very high current density up to 230 mA cm^−2^. However, most catalysts show poisoning of the surface via adsorbed intermediates. The poisoning of Pd is attributed to adsorbed intermediates such as CO and the degree of catalyst poisoning is related to the binding energy of those intermediates. Examples are presented that demonstrate the effectiveness of alloying on both the onset potential and the maximum achieved current (Table 3). The amount of catalyst poisoning for Pd-based materials can be predicted from DFT models, more specifically by analyzing the binding energy intermediates such as CO. By mapping the energetics of intermediates along a reaction mechanism, new materials can be predicted to perform better or worse than standard materials—presenting an opportunity for future in silico prediction of next generation Pd-based catalysts. The poisoning of FA electrocatalysts must be eliminated before formic acid/formate fuel cells are able to produce high power density. Thus, future research using a Pd catalyst for FAEOR should aim to reduce poisoning either by favoring a direct oxidation reaction mechanism, or through careful modulation of intermediate binding energies. Future publications should perform benchmark studies comparing the performance of novel electrocatalysts with known systems such as Pd disks or Pd on carbon for reliable results to be obtained. 

## 4. Pt and Pd-Free Materials for Formic Acid/Formate Electrooxidation

As discussed so far, from the early stages of research in hydrocarbon oxidation, palladium and platinum have been the most studied materials. Nevertheless, in spite of being successful as electrocatalysts for formic acid/formate oxidation, they are amongst the least abundant elements on earth. This fact, in addition to the CO-poisoning (especially problematic in the case of Pt), has triggered several efforts to find alternative electrocatalysts that can perform FA electrooxidation in an efficient way, and at the same time decrease the potential production costs of the fuel cells by using more abundant, cheaper elements. We can define two main strategies for the development of these alternative catalysts. The first strategy consists of reducing the load of Pt or Pd, by increasing the surface area, or alloying with other elements, as discussed in previous sections. The second strategy tries to fully replace Pt and Pd by studying other PGM materials, or even non-noble metals for FA oxidation. Many of these catalysts described in the literature show promising results, although examples of applications on fuel cells are scarce. 

As in previous sections, Table 4 summarizes the most relevant examples of catalysts for FA electrooxidation, and a brief discussion on their most relevant characteristics is offered in the text.

### 4.1. Bulk Materials

#### 4.1.1. Metals

Other metals have been studied as materials for anodes from the early developments of electrooxidation of organic molecules. The most studied alternative to Pt and Pd for FA oxidation is gold [54,58,60], although other metals like Rh [137] have also been shown to be active.

**Figure 10:**
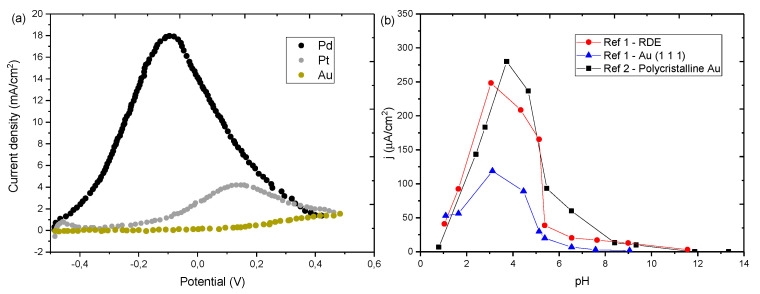
(**a**) CV forward scans of formic acid/formate oxidation on Pt, Pd and Au. Figure was redrawn with data taken from [54]; (**b**) dependence of current density with pH, at 1.0 V vs. RHE, for FA oxidation on Gold electrodes. Figure was redrawn with data taken from [58,60].

Gold electrodes show significantly lower current densities for FAEOR than Pt and Pd (see Figure 10a). However, gold offers some advantages like negligible CO-poisoning, and higher robustness than Pd and Pt [54,152]. The lack of CO poisoning in Au electrodes has prompted various studies on the mechanism of electrooxidation of FA. The absence of indirect oxidation reaction provides a simpler platform to understand the different parameters influencing the kinetics and efficiency of the reaction. Formic acid/formate oxidation reaction on gold is influenced by pH [58,60,152]. Current density shows its maximum value around pH 3–3.5, close to the FA pKa value, similar to what was observed with Pt catalysts above [46,55,58,60,99,100], and this occurs regardless of the gold surface structure (Figure 10b). The onset potential is constant below pH 5, where it starts shifting towards more positive potentials, approximately 60mV per pH unit [60].

Adsorbed formate has been proposed as the reactive intermediate since the 1970s [152]. More recently, detailed studies on the adsorption of formate on the surface of Au were carried out, with the purpose of elucidating the mechanism and finding ways to increase the efficiency of the oxidation reaction. ATR-SEIRAS measurements revealed that the only observed adsorbate on Au surface was formate on its bidentate configuration. Therefore, this was proposed as the main reactive intermediate, with the rate-limiting step of the oxidation being a bimolecular reaction between two neighbor formate molecules, followed by the oxidation of H_2_ [153]. However, this mechanism has been discarded based on other evidence, such as the pH dependence of the reaction and the absence of any proof of molecular hydrogen oxidation [58]. Moreover, the results obtained with electrochemical measurements and in situ STM, showed multiple configurations of adsorbed formate, presumably with different reactivities [154]. Monodentate formate or carboxylate were proposed as the most reactive of all the adsorbed species based on observations on the electrochemistry, although none of these species were detected. According to these authors, the rate limiting step would be the oxidation of the adsorbed monodentate formate.

As mentioned above with other catalytic systems, anions and other species in solution can also influence FA oxidation on Au electrodes. For example, phosphate buffers employed to fix the pH of the solution can bind active sites of a Au surface, competing with FA and therefore decreasing the current density observed [60]. This chemisorption is especially problematic at pH > 6, where the dominant phosphate species are HPO_4_^2−^ and PO_4_^3−^, which strongly bind to the Au surface through the oxygen atoms and block the active sites. This blocking effect is more intense in Au than in Pt electrodes, and therefore the choice of the pH and the electrolyte are crucial on application of gold.

Nevertheless, the adsorption of species on Au surface can also be used to boost the efficiency of the electrodes towards formic acid/formate oxidation. The suppression of strongly bound FA is considered a promising strategy for catalytic enhancement, and this can be achieved, for example, by addition of pyridine to the solution [136]. Pyridine can adsorb on Au surface in flat (by interaction through its pi-orbital) or perpendicular (through N atom) configuration. The flat configuration was found to induce a higher enhancement of FA oxidation on gold, thanks to the suppression of binding sites for bidentate formate. Therefore, these results agree with the mechanistic studies that point to monodentate adsorbed formate as the most reactive species.

The electrooxidation of methanol, ethanol and formic acid on Os electrodeposited on glassy carbon has been studied too [140]. MeOH and formic acid both oxidize at 0.62 and 0.77V, respectively. These oxidation processes appear in the same region as the electrooxidation of Os in the basic medium, indicating, as is widely accepted, that surface oxides are necessary for the electrooxidation of organic molecules. Electrochemical experiments with vibrational spectroscopy and linear potential sweep Ft-IR (LPS-FTIR) experiments show adsorption of CO following FA oxidation, which would indicate that on Os, the reaction follows the indirect pathway. CO adsorption on an Os surface is favorable, and the oxidation of chemisorbed CO requires high potentials. In general, the direct oxidation pathway is preferred to prevent CO poisoning of the electrodes, therefore it is unlikely that Os will find a practical application as oxidation catalyst of formic acid/formate.

#### 4.1.2. Metal Oxides

A perovskite-type oxide, La_0_._8_Sr_0.2_CoO_3_, has also been shown to be active as an anode material for FA oxidation [63]. It can catalyze the oxidation of formic acid via the direct oxidation pathway without any externally applied potential yielding a current density of approximately 2.2 mA cm^−2^, at 1.4 V vs. RHE. It has been shown to generate about five times more CO_2_ than Pt nanoparticles, as measured by gas chromatography. DFT calculations were carried out to get an insight on the mechanism of oxidation, showing that the lattice oxygen dissociates the FA molecule.

NiO on graphene oxide can be casted on Pt electrodes, also showing promising results for FA oxidation [51]. It shows higher catalytic activity (8.9 mA cm^−2^ at 0.6 V vs. RHE), lower onset potential (0.2 V vs. RHE) and better resistance towards CO poisoning than the unmodified Pt electrode (1.8 mA cm^−2^ at 1 V vs. RHE and onset potential of 0.4 V vs. RHE). The low poisoning is attributed to the effect of the residual oxygen functional groups present on the reduced graphene oxide, which would prevent the formation and adsorption of poisons.

Another example of active metal oxides for FA oxidation is IrO_2_ [141,155]. Electrodes based on IrO_2_ can be prepared by two methods: Thermal decomposition of an Ir precursor, or oxidation of an Ir anode. Direct-oxidation IrO_2_ anodes display faster kinetics towards formate oxidation [141]. Interestingly, the surface oxides involved in each case are different, and explain the observed difference in kinetics: for the IrO_2_ prepared by direct oxidation of Ir anode, the redox couple involved in the formic acid oxidation is the Ir(V)/Ir(IV), whereas for the IrO_2_ prepared by thermal decomposition, the surface redox couple Ir(VI)/Ir(IV) is the active one. The latter is the same redox pair involved in the oxygen evolution reaction, and therefore there is competition between the two reactions in this material.

#### 4.1.3. Tungsten Carbide

Although tungsten carbide has been known to be a promising electrocatalyst for oxidation of many organic molecules since the 1960s, it is not easy to trace detailed studies on its activity. In recent years, some groups have revisited this material as a catalyst for MeOH and H_2_ fuel cells [156]. 

One of the difficulties for the use of tungsten carbide is the importance of the preparation method for its performance as catalyst. Some of the advantages of this material for FA oxidation are its low tendency to poisoning, as well as its versatility to operate at different pHs, including neutral. Some mechanistic considerations were exposed by Palanker et al.; who did not observe any FA adsorption on the surface of the catalyst. Based on this, they suggested that the oxidation takes place via direct electron transfer from the molecules in solution to the electrode [143]. 

A tungsten carbide catalyst reached a record peak current of 60 mA cm^−2^ in a chronoamperometry at 0.3 V vs. RHE and 70 °C, after 3h of addition of formate [157]. Moreover, it was successfully applied as an anode in a microbial cell, in biological media, where it oxidizes formate with an onset potential of 0.43V vs. RHE [144]. 

#### 4.1.4. Co-Fe Prussian Blue

More recently, our group has reported a Co-Fe Prussian Blue derivative (CoFePB) as a formic acid/formate electrooxidation catalyst [53]. It was shown to be selective towards FA oxidation across a wide pH range (1–13), reaching very high Faradaic efficiencies (95 ± 5%), as measured in situ by mass spectrometry. 

**Figure 11:**
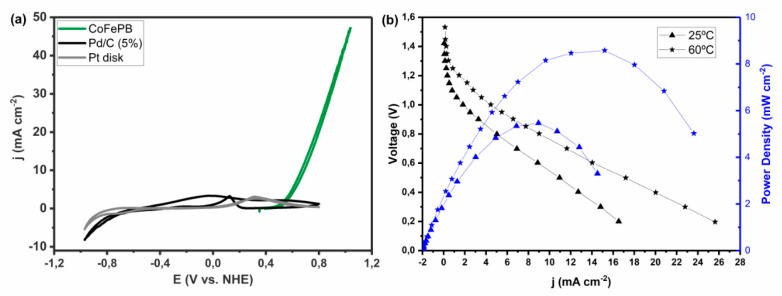
(**a**) Cyclic voltammetry of CoFePB compared to Pd and Pt; (**b**) polarization curve and power density of formate/Ce+4 fuel cell FC2 at 25 °C and 60 °C. Anode: CoFePB/FTO; Anotlyte: 2 M HCOO^−^ (pH 13); Cathode: Carbon felt; Catholyte: 1M HNO_3_ (pH 0). Figures reprinted with permission, copyright to (**a**) JACS and (**b**) RSC.

Moreover, the good electrode stability and the lack of CO poisoning, as supported by voltammetry and EIS measurements, make it possible to reach much higher current densities at high potentials than Pd and Pt catalysts (Figure 11). For instance, at pH 5, CoFePB reaches c.a. 100 mA cm^−2^ at 1.6 V vs. RHE with a scan rate of only 5 mV s^−1^. CoFePB is a promising candidate for application in sensing or fuel cells if the electrode configuration is improved to decrease its overpotential values, which are among the highest ones in the literature. Another way to take advantage of this and other catalysts reviewed herein showing high FA oxidation overpotentials, for fuel cell application, is to operate the anode in alkaline media while operating a strong oxidant as cathode in acidic media.

In fact, our group also prepared a proof-of-concept formate/Ce^4+^ fuel cell that, without being optimized, returned a stable maximum power output of 8.6 mW cm^−2^ (Figure 11). This is the first reported case of a fully non-platinum group metals (PGM) direct formate fuel cell (including both anode and cathode) [23]. 

### 4.2. New Trends in Formate Oxidation Catalysts

The progress in the search for cheap, robust and efficient electrocatalysts for anodes in direct FA fuel cells is taking other directions than the pure discovery and application of bulk materials.

#### 4.2.1. Nanoparticles

Nanostructured materials can have increased catalytic activity because of the greater surface area of the electrode material, increased concentration of defect sites and other changes that occur when transitioning from bulk to nanostructured materials. 

Rhodium nanostructures are attractive materials that may be interesting for fuel cell applications due to their high tolerance vs. CO poisoning. They also display size-dependent electrocatalytic properties. A recent paper studied the performance of nanostructures formed by the assembly of Rh nanoparticles and found enhanced electrocatalytic activity towards formic acid oxidation, plus a negative shift in the onset potential and higher current density than that corresponding to bulk Rh [138]. 

Another work explored the fabrication of graphene nanoplatelet (GNP)-supported Ir–Zn catalysts [146]. These catalysts were prepared in various Ir:Zn ratios by H_2_ reduction. The optimum ratio was found to be 50:50, where the highest electrochemical performance for formic acid electrooxidation was found, with an overpotential as low as 0.2 V, comparable to those obtained with Pd-based catalysts. The role of Zn in the nanoparticles was found to be adsorbed into the active sites of Ir, in a way that it can enhance the activity of the catalyst by facilitating the direct pathway reaction. However, when there is a proportion of Zn above 50%, it blocks all the active sites of Ir and the catalyst loses activity.

#### 4.2.2. Polymer Composites

Polymer nanocomposites containing catalysts are of interest for many areas of research because they frequently exhibit unexpected hybrid properties, synergically derived from both components. The field of FA fuel cells is not an exception in this sense, and a few attempts were made.

A composite of gold clusters and polyindole [147] showed better CO tolerance capability, with excellent activity and stability compared to Au commercial electrodes. This is due to a synergic effect between the gold nanoclusters and the electron-rich centers of the polymer.

Similar to gold nanoclusters, some metal oxides were successfully embedded into polymer matrices and proved their electrocatalytic properties towards formic acid oxidation [148,149]. Namely, SnO_2_ and MnO_2_ were successful in this sense, although in the case of MnO_2_, it was shown that polyaniline in its emeraldine form is the most probable electrocatalyst. Even though the cyclic voltammetry of the MnO_2_-polyaniline composite did not show activity towards FA oxidation, this is one of the few examples that can be found in the literature of a working FAFC with a non-PGM catalyst in the anode. This cell showed an open circuit voltage of 0.55V and a maximum power density of 3 mW cm^−2^ at 10 mA cm^−2^ at room temperature [148]. 

#### 4.2.3. Single Atom Catalysts

Nitrogenated porous graphitic carbon materials were utilized as templates for atomically disperse metal catalysts [150,151,158]. Very often, the single-atom properties are different to the bulk material, opening unexplored possibilities. This strategy was recently reported for Rh and Ir in FA oxidation. Interestingly, the activity of these two metals, which is very low, is greatly enhanced when they are incorporated as single atoms into graphitic porous materials. For example, a single-atom Ir electrocatalyst on nitrogenated graphene (Ir_1_/CN) exhibits excellent mass activity, 12.9 A mg^−1^, 16 and 19 times greater than those of Pd/C and Pt/C, respectively. First-principle density functional theory revealed that the origin of the properties of Ir_1_/CN, aside from the modified electronic structure, is related to the spatial isolation of iridium sites [150]. For the Rh analogous material, the mass activity (16.1 A mg^−1^) is 28- and 67-fold higher than that of state-of-the-art Pd/C and Pt/C, respectively, with similar overpotentials, around 0.2 V vs. RHE [151,158]. 

### 4.3. Benchmarking Pd and Pt-free Formic Acid/Formate Electrooxidation Catalysts

Due to the wide variety of materials included in this category, it is hard to suggest one example that can serve as reference for comparison of new materials. From our point of view, it is always advisable to compare the activity of new catalysts with classical ones based on Pd and Pt (see tables of Pd and Pt catalysts), and, beyond that, our additional suggestions are summarized in Table 5. We have chosen three catalysts: (i) An Au disk, because it is a widely studied material, and commercially available. However, one must take into account the pH range in which Au electrodes are active (see above). (ii) We think CoFePB is a promising candidate because it offers high FA oxidation current density and robustness at any pH. As an example, we have included in the table its performance at pH 5 and 13 with a given HCOOH concentration, but data for other conditions can be found in reference [53]. Finally, we note the catalyst based on Rh single atoms on nitrogen-doped carbon [151], since it is one of the best-performing catalyst in terms of both the onset potential and the peak current density, among the Pt- and Pd-free catalysts.

## 5. Conclusions

In this review, we have benchmarked catalysts for formic acid/formate (FA) electrooxidation reaction. This reaction is becoming increasingly attractive especially as a primary way to directly extract energy from FA as a carbon-neutral energy carrier. Apart from controversial reaction mechanism considerations, which have been thoroughly studied using different in situ spectroscopy techniques, this review is focused on the selection of anode materials and operation conditions for direct formic acid/formate fuel cells applications. Since the early 1960s of the twentieth century, many materials have been experimentally and theoretically evaluated as possible catalysts and tested with several techniques in different experimental conditions. This calls for an effort for a deep review of the field, which can be a guideline for choosing the most proper reference materials and standardizing the testing protocols. In our opinion, this would mark a breakthrough towards the development of more active and durable catalysts and a turning point in the exploitation of FA as fuel.

Our first consideration is regarding the wide variability found in the literature with respect to the experimental variables considered and the experimental procedures followed, but also to the way the data are presented. Cyclic voltammetry (CV) is the most used and accessible electrochemistry technique, giving much useful information for catalyst screening. For this reason, it has been the selected tool to evaluate catalytic performance, and we have made an effort to normalize and systematically analyze the data.

Pt and Pd, pure with different atomic structures/crystallinities or in combination with other materials, are the most investigated FA electrooxidation catalysts. They are characterized by a low onset potential (*E_onset_*), generally 0.1–0.2 V vs. RHE and 0.2–0.3V vs. RHE for Pd and Pt, respectively. However, both catalysts suffer from severe poisoning especially from CO species, which limit the obtained current density and stability. The lack of stability due to the poisoning is a serious limitation for practical applications and have to be addressed. Although the reaction mechanism and consequently the poisoning effect are not yet clear, some conclusions can be drawn: I) The poisoning effect is more relevant for Pt than Pd, especially in alkaline media; II) CO poisoning in Pd is more related to CO_2_ reduction at low potential; III) in Pt, CO is an intermediate in the indirect pathway; IV) the introduction in the catalyst surface of oxygen atoms could facilitate CO oxidation; and lastly, V) alloying of Pd and Pt with other elements reduces the catalyst deactivation either by changing the binding energy of CO and other intermediates or by enhancing the CO oxidation rate or by creating an alternative reaction pathway. Alloying is thus a way towards the synthesis of more stable materials, which has to be taken into account together with the suppression of the indirect pathway.

Elements other than Pt and Pd for FA oxidation have been investigated, with Au being the most studied. Generally, these catalysts have higher onset potential (˃0.3 V vs. RHE) but are typically more stable/less susceptible to poisoning and therefore can achieve a higher current density (e.g., CoFe Prussian Blue) than catalysts based on Pt and Pd.

In all cases, catalytic performances are affected by the testing conditions, such as pH, formic acid/formate concentration, electrolytes, the presence of a buffer, temperature, and, most importantly, the nature of the electrocatalyst. Concerning the pH, a generally volcano-type behavior is observed with better performances generally obtained at pH close to neutrality or to the formic acid/formate pKa. This suggests a pH-dependent reaction pathway with different intermediates involved. Optimum formic acid/formate concentrations are close to 1 M and generally in the range of 0.25–2 M. Caution should be addressed in the use of buffer solutions. Although buffers are advantageous in terms of pH control, the presence of species like phosphate could strongly bind to the catalyst surface, being detrimental to the catalytic reaction by blocking active sites and reducing current density—hence, the use of an electrolyte such as H_2_SO_4_ or HClO_4_ is encouraged in acidic conditions, while KOH is the best in alkaline conditions. Another important parameter is the temperature affecting both thermodynamic and kinetic poisoning. However, tests are generally performed at room temperature. Of great interest would be increasing the temperature to 60–80 °C, the temperature of actual fuel cell operation. Another important factor to investigate in more detail would be the effect of the ionomer in the electrode that could affect the reaction through both the acid/alkaline and ion exchange intrinsic properties. Another key parameter is the Faradaic efficiency, to determine the catalyst selectivity. This parameter, although often overlooked, should also be investigated as fundamental for other applications such as sensing. This can be easily evaluated by coupling the electrochemical cell to a mass spectrometer, for example, to monitor the CO_2_ evolution. Finally, another technique that has been carried out in a few cases is the ICP-MS in order to monitor the metal dissolution during the electrochemical tests. This procedure is very useful to monitor the stability of the metal catalysts under the studied operation conditions (i.e., pH, potential, temperature).

In Table 5, we have suggested some reference electrodes and standard testing conditions for both Pd, Pt and Pt- and Pd-free materials. Based on what we previously mentioned, we believe that Pd(40%)/Vulcan carbon should be the benchmark material to compare catalytic activity of new catalysts due to the very low overpotential and high maximum current density in both acid (0.1 V vs. RHE, 140 mA cm^−2^) and alkaline media (0.2 V vs. RHE, 102 mA cm^−2^). Moreover, its easy preparation procedure makes it a perfect candidate for catalytic activity screenings.

We suggest the development of more catalysts with high non-porosity or catalysts with high dispersion and low metal loading. Finally, we also suggest CoFe Prussian Blue/SnO_2_:F as a non-PGM benchmark catalyst due to the outstanding stability and poisoning resistance, which allow it to reach a very high current density (97.3 mA cm^−2^ at 1.6 V vs. RHE) with a very high faradaic efficiency (95 ± 5%). Despite the very high onset potential (1.2 V vs. RHE), it is one of the most promising noble metal-free catalyst for FA electrooxidation, which has been also tested in a direct formate fuel cell.

## Figures and Tables

**Table 1:** Oxidant reactions for use in direct formic acid/formate fuel cell and theoretical cell voltages.

Oxidant	Reaction	Thermodynamic Potential, E^0^ vs. NHE (V) ^i^	Theoretical Fuel Cell Voltage (V) ^ii^	
			With Acid Anode (HCOOH Oxidation at pH 0)	With Alkaline Anode (HCOO^−^ Oxidation at pH 14)
O_2_/Air	O_2_ + 4H^+^ + 4e^−^ → 2H_2_O	1.229	1.449	2.275
K_2_CrO_7_	Cr_2_O_7_^2−^ + 14H^+^ + 6e^−^ → 2Cr^3+^ + 7H_2_O	1.360	1.580	2.406
HClO	HClO + H^+^ + 2e^−^ → Cl^−^ + H_2_O	1.482	1.702	2.528
	HClO + H^+^ + e^−^ → 0.5Cl_2_ + H_2_O	1.611	1.831	2.657
KMnO_4_	MnO_4_^−^ + 8H^+^ + 5e^−^ → Mn^2+^ + 4H_2_O	1.507	1.727	2.553
	MnO_4_^−^ + 4H^+^ + 3e^−^ → MnO_2_ + 2H_2_O	1.679	1.899	2.725
Ce(NH_4_)_2_(NO_3_)_6_	Ce^4+^ + e^−^ → Ce^3+^	1.720	1.940	2.766
H_2_O_2_	H_2_O_2_ + 2H^+^ + 2e^−^ → 2H_2_O	1.776	1.996	2.822
K_2_S_2_O_8_	S_2_O_8_^2−^ + 2H^+^ + 2e^−^ → 2HSO_4_^−^	2.123	2.343	3.169

^i^ Standard potentials from Ref. [18]. ^ii^ Cell voltages calculated by considering a formic acid oxidation standard potential of −0.22 V vs. NHE.

**Table 2:** Benchmarking of Pt-based electrocatalysts for formic acid/formate oxidation in half-cell configuration at ambient conditions, in acidic and basic media, in terms of onset potential (*E_onset_*), maximum current density (*j_max_*, normalized by electrode geometric area) obtained on anodic peaks (at a potential *E_peak_*) and current density (*j*) obtained at given potentials.

Ref.	Catalyst	Experimental Conditions ^i^	*E_onset_* (V vs. RHE) ^ii^	*j_max_* (mA cm^−2^) at *E_peak_* (V vs. RHE) ^ii^	*j* (mA cm^−2^) ^ii^ at			
					1.00 V vs. RHE	1.20 V vs. RHE	1.40 V vs. RHE	1.60 V vs. RHE
[40]	Pt disk	0.2 M HCOOH + 1 M HClO_4_ (pH ≈ 0) 20 mV s^−1^	0.2	1.0 mA cm^−2^ (0.9 V vs. RHE)	0.4	0.2	0.9	-
		0.2 M HCOOH + 1 M NaOH (pH ≈ 14.0) 20 mV s^−1^	0.3	0.1 mA cm^−2^ (0.5 V vs. RHE)	<0.1	<0.1	<0.1	-
[51]	Pt disk	0.3 M HCOOH (pH 3.5) 100 mV s^−1^	0.4	1.8 mA cm^−2^ (1.0 V)	1.7	-	-	-
[52]	Pt disk	1 M HCOONa + 0.1 M H_2_SO_4_ (pH 4.5) 50 mV s^−1^	0.2	2.5 mA cm^−2^ (0.6 V vs. RHE)	1.9	1.8	-	-
[53]	Pt disk	0.4 M HCOOH + 1 M KNO_3_ (pH 5) 5 mV s^−1^	0.3	0.5 mA cm^−2^ (1.4 V vs. RHE)	0.3	0.3	0.4	0.3
		0.4 M HCOOH + 1 M KNO_3_ (pH 13) 5 mV s^−1^	0.5	3.1 mA cm^−2^ (1.1 V vs. RHE)	2.1	2.0	0.9	-
[54]	Pt disk	0.1 M HCOOK + 0.2 M K_2_SO_4_ (pH ≈ 8.4) 50 mV s^−1^	0.5	4.3 mA cm^−2^ (0.9 V vs. RHE)	2.7	1.5	-	-
[55]	Pt rotating disk	0.1 M HCOOH + 0.2 M KPi (pH 3.7) 50 mV s^−1^, 1000 rpm	0.5	2.9 mA cm^−2^ (0.9 V vs. RHE)	0.9	0.4	0.3	-
[56]	Pt rotating disk	0.02 M HCOONa + 0.2 M KPi (pH 4.2) 20 mV s^−1^, 1000 rpm	0.3	8.5 mA cm^−2^ (0.9 V vs. RHE)	7.7	4.5	-	-
[57]	Pt net	0.1 M HCOOH + 0.5 M H_2_SO_4_ (pH ≈ 0.3) 100 mV s^−1^	0.3	3.1 mA cm^−2^ (0.9 V vs. RHE)	1.5	0.4	2.9	-
[58]	Pt bead	0.1 M HCOOH + 0.5 M Na_2_SO_4_ (pH 3.6) 50 mV s^−1^	0.5	5.4 mA cm^−2^ (0.9 V vs. RHE)	2.4	1.0	0.6	-
[59]	Pt(111)	0.1 M HCOOH + 0.1 M HClO_4_ (pH ≈ 1.0) 50 mV s^−1^	0.3	1.5 mA cm^−2^ (0.6 V vs. RHE)	<0.1	-	-	-
[60]	Pt(111)	0.05 M HCOONa + 0.2 M NaPi (pH 5.1) 10 mV s^−1^	0.3	2.4 mA cm^−2^ (0.6 V vs. RHE)	-	-	-	-
[61]	Pt(111)	0.1 M HCOOH + 0.5 M H_2_SO_4_ (pH ≈ 0.3) 50 mV s^−1^	0.3	2.2 mA cm^−2^ (0.6 V vs. RHE)	-	-	-	-
		0.1 M HCOOH + 0.1 M HClO_4_ (pH ≈ 1.0) 50 mV s^−1^	0.3	2.8 mA cm^−2^ (0.5 V vs. RHE)	-	-	-	-
[62]	Pt nanoparticles	0.5 M HCOOH + 0.1 M H_2_SO_4_ (pH ≈ 1.0) 50 mV s^−1^	0.2	10 mA cm^−2^ (0.8 V vs. RHE)	3.4	6.1	8.3	-
[63]	Pt nanoparticles	2.1 M HCOOH + 0.5 M KNO_3_ (pH ≈ 1.7) 40 mV s^−1^	0.5	55.0 mA cm^−2^ (1.2 V vs. RHE)	45.0	48.0	36.0	-
[64]	Pt nanoparticles (Pt black)	0.5 M HCOOH + 0.5 M H_2_SO_4_ (pH ≈ 0.3) 50 mV s^−1^	0.2	0.7 mA cm^−2^ (0.6 V vs. RHE)	0.5	0.4	-	-
		0.5 M HCOOK + 0.5 M KOH (pH ≈ 13.7) 50 mV s^−1^	0.2	0.2 mA cm^−2^ (0.5 V vs. RHE)	<0.1	<0.1	-	-
[65]	Pt nanoparticles (Pt black)	1 M HCOOK + 1 M KOH (pH ≈ 14.0) 50 mV s^−1^	0.3	2.6 mA cm^−2^ (0.5 V vs. RHE)	<1.0	-	-	-
[52]	Polyaniline/Pt disk	1 M HCOONa + 0.1 M H_2_SO_4_ (pH 4.5) 50 mV s^−1^	0.2	23.0 mA cm^−2^ (0.8 V vs. RHE)	8.6	4.5	6.8	-
[66]	Pt nanoparticles/Polyaniline/SBA-15	0.5 M HCOOH + 0.5 M H_2_SO_4_ (pH ≈ 0.3) 50 mV s^−1^	0.3	33.2 mA cm^−2^ (1.0 V vs. RHE)	32.9	15.6	-	-
[67]	Pt(20%)/C	0.5 M HCOOH + 0.5 M H_2_SO_4_ (pH ≈ 0.3) 50 mV s^−1^	0.2	13.1 mA cm^−2^ (1.0 V vs. RHE)	12.0	10.0	-	-
[68]	Pt(20%)/C	0.5 M HCOOH + 0.1 M HClO_4_ (pH ≈ 1) 20 mV s^−1^	0.3	13.5 mA cm^−2^ (1.0 V vs. RHE)	9.9	-	-	-
[69]	Pt(20%)/Vulcan carbon	0.25 M HCOOH + 0.1 M HClO_4_ (pH ≈ 1.0) 50 mV s^−1^	0.3	4.4 mA cm^−2^ (0.9 V vs. RHE)	2.7	-	-	-
[70]	Pt(40%)/Vulcan carbon	1 M HCOOH + 1 M H_2_SO_4_ (pH ≈ 0) 20 mV s^−1^	0.2	110.0 mA cm^−2^ (1.1 V vs. RHE)	96.0	58.0	78.0	-
		1 M HCOOK + 1 M KOH (pH ≈ 14.0) 20 mV s^−1^	0.4	14.6 mA cm^−2^ (0.6 V vs. RHE)	3.4	3.4	3.7	9.2
[71]	Pt(50%)/C	0.5 M HCOOH + 0.5 M KOH (pH ≈ 13.7) 50 mV s^−1^	0.8	33.9 mA cm^−2^ (1.2 V vs. RHE)	18.5	33.0	13.7	8.0
[72]	Pt nanoparticles/Vulcan carbon	0.5 M HCOOH + 0.5 M H_2_SO_4_ (pH ≈ 0.3) 50 mV s^−1^	0.3	5.2 mA cm^−2^ (1.0 V vs. RHE)	5.2	6.4	-	-
[62]	Pt nanoparticles/Carbon nanoparticles	0.5 M HCOOH + 0.1 M H_2_SO_4_ (pH ≈ 1.0) 50 mV s^−1^	0.1	38.9 mA cm^−2^ (0.9 V vs. RHE)	23.4	23.7	32.5	-
	Pt nanoparticles/Reduced graphene oxide	0.5 M HCOOH + 0.1 M H_2_SO_4_ (pH ≈ 1.0) 50 mV s^−1^	0.2	27.7 mA cm^−2^ (0.9 V vs. RHE)	9.7	10.4	12.7	-
[73]	Pt nanoparticles/ TiO_2_ nanotubes	0.5 M HCOOH + 0.5 M H_2_SO_4_ (pH ≈ 0.3) 10 mV s^−1^	0.8	14.0 mA cm^−2^ (1.0 V vs. RHE)	14.0	4.4	-	-
[74]	Pt monolayer/Ru(0001)	0.5 M HCOOH + 0.1 M HclO_4_ (pH ≈ 1.0) 50 mV s^−1^	0.2	1.1 mA cm^−2^ (0.9 V vs. RHE)	0.9	-	-	-
	Pt monolayer/Rh(111)	0.5 M HCOOH + 0.1 M HClO_4_ (pH ≈ 1.0) 50 mV s^−1^	0.2	1.5 mA cm^−2^ (1.0 V vs. RHE)	1.4	-	-	-
	Pt monolayer/Pd(111)	0.5 M HCOOH + 0.1 M HClO_4_ (pH ≈ 1.0) 50 mV s^−1^	0.2	0.6 mA cm^−2^ (1.0 V vs. RHE)	0.4	-	-	-
	Pt monolayer/Au(111)	0.5 M HCOOH + 0.1 M HClO_4_ (pH ≈ 1.0) 50 mV s^−1^	0.2	7.3 mA cm^−2^ (0.6 V vs. RHE)	4.1	-	-	-
[75]	Pt disk + 1 × 10^−3^ M Cd^2+^ (in electrolyte)	0.265 M HCOOH + 1 M HClO_4_ (pH ≈ 0) 50 mV s^−1^	0.2	7.2 mA cm^−2^ (0.5 V vs. RHE)	1.0	0.9	3.3	-
	Pt disk + 5 × 10^−4^ M Ti^+^ (in electrolyte)	0.265 M HCOOH + 1 M HClO_4_ (pH ≈ 0) 50 mV s^−1^	0.1	20.7 mA cm^−2^ (0.5 V vs. RHE)	1.1	-	-	-
	Pt disk + 1 × 10^−3^ M Bi^3+^ (in electrolyte)	0.265 M HCOOH + 1 M HClO_4_ (pH ≈ 0) 50 mV s^−1^	0.5	46.0 mA cm^−2^ (0.8 V vs. RHE)	1.1	1.0	1.9	-
	Pt disk + 1 × 10^−3^ M Pb^2+^ (in electrolyte)	0.265 M HCOOH + 1 M HClO_4_ (pH ≈ 0) 50 mV s^−1^	0.2	70.2 mA cm^−2^ (0.5 V vs. RHE)	1.3	1.5	3.7	-
[76]	PtRu	0.1 M HCOOH + 0.1 M HClO_4_ (pH ≈ 1.0) 100 mV s^−1^	−0.1	2.2 mA cm^−2^ (0.6 V vs. RHE)	2.7	-	-	-
[77]	PtRu	0.05 M HCOOH + 0.1 M HClO_4_ (pH ≈ 1.0) 10 mV s^−1^	0.2	5.1 mA cm^−2^ (0.8 V vs. RHE)	2.9	1.7	-	-
[70]	Pt_1_Ru_1_(40%)/Vulcan carbon	1 M HCOOH + 1 M H_2_SO_4_ (pH ≈ 0) 20 mV s^−1^	0.3	145.0 mA cm^−2^ (1.0 V vs. RHE)	145.0	44.0	49.0	-
[78]	PtAu/C	1 M HCOOH + 0.5 M H_2_SO_4_ (pH ≈ 0.3) 20 mV s^−1^	0.1	4.0 mA cm^−2^ (0.6 V vs. RHE)	1.8	1.3	-	-
[68]	PtCu/C	0.5 M HCOOH + 0.1 M HClO_4_ (pH ≈ 1) 20 mV s^−1^	0.3	30.8 mA cm^−2^ (1.1 V vs. RHE)	19.0	-	-	-
	Pt/SnO_2_/C	0.5 M HCOOH + 0.1 M HClO_4_ (pH ≈ 1) 20 mV s^−1^	0.2	32.8 mA cm^−2^ (1.1 V vs. RHE)	14.3	-	-	-
[69]	Pt(20%)Bi/Vulcan carbon	0.25 M HCOOH + 0.1 M HClO_4_ (pH ≈ 1.0) 50 mV s^−1^	0.1	46.1 mA cm^−2^ (0.8 V vs. RHE)	3.0	-	-	-
[79]	PtFe nanoparticles	0.1 M HCOOH + 0.1 M HClO_4_ (pH ≈ 1.0) 100 mV s^−1^	0.2	32.3 mA cm^−2^ (1.3 V vs. RHE)	14.3	22.4	30.2	-
[67]	Pt_0.05_Au nanowires	0.5 M HCOOH + 0.5 M H_2_SO_4_ (pH ≈ 0.3) 50 mV s^−1^	0.2	33.5 mA cm^−2^ (0.6 V vs. RHE)	10.0	11.0	-	-
[66]	PtCo nanoparticles	0.5 M HCOOH + 0.5 M H_2_SO_4_ (pH ≈ 0.3) 50 mV s^−1^	0.4	19.5 mA cm^−2^ (1.0 V vs. RHE)	17.4	9.1	-	-
	PtCo nanoparticles/Polyaniline/SBA–15	0.5 M HCOOH + 0.5 M H_2_SO_4_ (pH ≈ 0.3) 50 mV s^−1^	0.3	63.8 mA cm^−2^ (1.1 V vs. RHE)	46.8	39.4	-	-
[65]	PtAg alloy nanoballoon nanoassembly	1 M HCOOK + 1 M KOH (pH ≈ 14.0) 50 mV s^−1^	0.2	32.6 mA cm^−2^ (0.7 V vs. RHE)	<2.0	-	-	-
[80]	Pt(1.3 at.%)AuCu dealloyed	2 M HCOOH + 0.1 M HClO_4_ (pH ≈ 0.9) 50 mV s^−1^	0.2	54.8 A mg_Pt_^−1^ (1.1 V vs. RHE) ^iii^	52.1 A mg_Pt_^−1 iii^	1.4 A mg_Pt_^−1 iii^	-	-
	Pt(2.6 at.%)AuCu dealloyed	2 M HCOOH + 0.1 M HClO_4_ (pH ≈ 0.9) 50 mV s^−1^	0.2	-	28.0 ^iii^	35.8 ^iii^	-	-

^i^ Approximate pH values estimated from the acid/base concentrations, when they are not specified in the articles. ^ii^ Approximate values estimated from the experimental details and the voltammograms (forward scan) shown in the articles. ^iii^ It is not possible to transform these values into mA cm^−2^.

**Table 3:** Benchmarking of Pd-based electrocatalysts for formic acid/formate oxidation in half-cell configuration at ambient conditions, in acidic and basic media, in terms of onset potential (*E_onset_*), maximum current density (*j_max_*, normalized by electrode geometric area) obtained on anodic peaks (at a potential *E_peak_*) and current density (*j*) obtained at given potentials.

Ref.	Catalyst	Experimental Conditions ^i^	*E_onset_* (V vs. RHE) ^ii^	*j_max_* (mA cm) at *E_peak_* (V vs. RHE) ^ii^	*j* (mA cm^−2^) ^ii^ at			
					1.00 V vs. RHE	1.20 V vs. RHE	1.40 V vs. RHE	1.60 V vs. RHE
[30]	Pd disk	1.0 M HCOOH + 0.5 M H_2_SO_4_ (pH ≈ 0.3) 70 mV s^−1^	N/A	2.8 mA/cm^−2^ (0.36 V vs. RHE)	0.12	0.087	0.094	-
[110]	Pd foil	0.01 M HCOOH + 0.5 M HClO_4_ (pH ≈ 0.3) 50 mV s^−1^	0.2	3.2 mA/cm (0.3 V vs. RHE)	0.11	0.11	0.16	1.07
[111]	Pd(100)	0.1 M HCOOH + 0.1 M HClO_4_ (pH ≈ 1.0) 20 mV s^−1^	0.1	19.7 mA cm (0.77 V vs. RHE)	<1.0	<1.0	-	-
	Pd(110)	0.1 M HCOOH + 0.1 M HClO_4_ (pH ≈ 1.0) 20 mV s^−1^	0.1	11.0 mA cm (0.52 V vs. RHE)	<1.0	<1.0	-	-
	Pd(111)	0.1 M HCOOH + 0.1 M HClO_4_ (pH ≈ 1.0) 20 mV s^−1^	0.2	4.8 mA cm (0.5 V vs. RHE)	<1.0	<1.0	-	-
[112]	Pd nanocubes	0.5 M HCOOH + 0.1 M HClO_4_ (pH ≈ 1.0) 50 mV s^−1^	0.2	10.1 mA cm (0.54 V vs. RHE)	1.0	-	-	-
	Pd nanooctahedra	0.5 M HCOOH + 0.1 M HClO_4_ (pH ≈ 1.0) 50 mV s^−1^	0.2	6 mA cm^−2^ (0.47 V vs. RHE)	0.45	-	-	-
	Pd nanoicosahedrons	0.5 M HCOOH + 0.1 M HClO_4_ (pH ≈ 1.0) 50 mV s^−1^	0.2	10.4 mA cm^−2^ (0.46 V vs. RHE)	0.32	-	-	-
[71]	Pd black	0.5 M HCOOH + 0.5 M KOH (pH ≈ 13.7) 50 mV s^−1^	0.8	27.3 mA cm^−2^ (1.2 V vs. RHE)	13.6	26.4	12.5	6.0
[113]	Pd black	0.5 M HCOOH + 0.5 M H_2_SO_4_ (pH ≈ 0.3) 50 mV s^−1^	0.1	5.4 mA cm^−2^ (0.5 V vs. RHE)	<1.0	<1.0	-	-
[114]	Pd (nanoporous)	0.5 M HCOOH + 0.5 M H_2_SO_4_ (pH ≈ 0.3) 10 mV s^−1^	0.1	232 mA cm^−2^ (0.6 V vs. RHE)	103.0	-	-	-
[53]	Pd(5%)/Vulcan carbon	0.4 M HCOOH + 1 M KNO_3_ (pH 5) 5 mV s^−1^	0.1	0.6 mA cm^−2^ (0.6 V vs. RHE)	0.5	0.7	1.4	3.4
		0.4 M HCOOH + 1 M KNO_3_ (pH 13) 5 mV s^−1^	0.2	3.3 mA cm^−2^ (0.7 V vs. RHE)	2.4	2.2	1.9	-
[115]	Pd/C	1 M HCOOK + 1 M KOH (pH ≈ 14.0) 50 mV s^−1^	0.1	108.8 mA cm^−2^ (0.7 V vs. RHE)	14.6	-	-	-
[116]	Pd(20%)-H/Vulcan carbon	0.5 M HCOOK + 1 M KOH (pH ≈ 14.0) 20 mV s^−1^	0.2	71.0 mA cm^−2^ (0.8 V vs. RHE)	<1.0	<1.0	-	-
[117]	Pd(20%)/C	1 M HCOONa + 1 M NaOH (pH ≈ 14.0) 20 mV s^−1^	0.2	40.0 mA cm^−2^ (0.8 V vs. RHE)	28.0	25.0	24.0	22.8
[118]	Pd(25%)/Vulcan carbon	1 M HCOOK + 1 M KOH (pH ≈ 14.0) 50 mV s^−1^	0.2	4.6 mA cm^−2^ (0.7 V vs. RHE)	<0.5	<0.5	-	-
[119]	Pd(30%)/Vulcan carbon	0.1 M HCOOK + 1 M KOH (pH ≈ 14.0) 20 mV s^−1^	0.2	14.7 mA cm^−2^ (0.5 V vs. RHE)	<1.0	<1.0	-	-
[70]	Pd(40%)/Vulcan carbon	1 M HCOOH + 1 M H_2_SO_4_ (pH ≈ 0) 20 mV s^−1^	0.1	140.0 mA cm^−2^ (0.8 V vs. RHE)	60.0	16.0	20.0	-
		1 M HCOOK + 1 M KOH (pH ≈ 14.0) 20 mV s^−1^	0.2	102.0 mA cm^−2^ (1.0 V vs. RHE)	101.1	65.8	65.5	27.1
[120]	Pd/Vulcan carbon	1 M HCOOK + 1 M KOH (pH ≈ 14.0) 50 mV s^−1^	0.4	23.0 mA cm^−2^ (0.8 V vs. RHE)	<3.0	-	-	-
[121]	Pd/Vulcan carbon	0.5 M HCOOH + 0.5 M H_2_SO_4_ (pH ≈ 0.3) 50 mV s^−1^	0.1	3.6 mA cm^−2^ (0.3 V vs. RHE)	0.5	0.7	-	-
	Pd/Graphene	0.5 M HCOOH + 0.5 M H_2_SO_4_ (pH ≈ 0.3) 50 mV s^−1^	0.1	7.7 mA cm^−2^ (0.4 V vs. RHE)	2.4	2.0	-	-
	Pd/N-graphene/CNT	0.5 M HCOOH + 0.5 M H_2_SO_4_ (pH ≈ 0.3) 50 mV s^−1^	0.1	17.6 mA cm^−2^ (0.4 V vs. RHE)	4.2	3.7	-	-
[122]	Pd(13%)/Reduced graphene oxide	0.5 M HCOOH + 0.5 M H_2_SO_4_ (pH ≈ 0.3) 50 mV s^−1^	0.1	13.2 mA cm^−2^ (0.4 V vs. RHE)	1.4	1.5	-	-
[117]	Pd(20%)/Reduced graphene oxide	1 M HCOONa + 1 M NaOH (pH ≈ 14.0) 20 mV s^−1^	0.2	57.0 mA cm^−2^ (0.8 V vs. RHE)	36.0	15.0	6.4	6.6
[73]	Pd/TiO_2_	0.5 M HCOOH + 0.5 M H_2_SO_4_ (pH ≈ 0.3) 10 mV s^−1^	0.1	13.0 mA cm^−2^ (0.4 V vs. RHE)	2.3	1.2	-	-
[123]	Pd-H wire	0.24 M HCOONa + 0.24 M NaOH (pH ≈ 13.4) 50 mV s^−1^	0.1	15.1 mA cm^−2^ (0.8 V vs. RHE)	1.2	1.0	-	-
[74]	Pd(111)	0.5 M HCOOH + 0.1 M HClO_4_ (pH ≈ 1.0) 50 mV s^−1^	0.1	2.9 mA cm^−2^ (0.47 V vs. RHE)	2.0	-	-	-
	Pd monolayer/Ir(111) ^iii^	0.5 M HCOOH + 0.1 M HClO_4_ (pH ≈ 1.0) 50 mV s^−1^	0.1	4.3 mA cm^−2^ (0.4 V vs. RHE)	1.5	-	-	-
	Pd monolayer/ Au(111) ^iii^	0.5 M HCOOH + 0.1 M HClO_4_ (pH ≈ 1.0) 50 mV s^−1^	0.1	15 mA cm^−2^ (0.7 V vs. RHE)	5.9	-	-	-
	Pd monolayer/ Pt(111) ^iii^	0.5 M HCOOH + 0.1 M HClO_4_ (pH ≈ 1.0) 50 mV s^−1^	0.1	55 mA cm^−2^ (0.97 V vs. RHE)	4.6	-	-	-
[124]	PdBi nanoparticles	0.1 M HCOOH + 0.5 M H_2_SO_4_ (pH ≈ 0.3) 50 mV s^−1^	0.2	7.6 mA cm^−2^ (0.4 V vs. RHE)	3.5	2.0	-	-
	PdCd nanoparticles	0.1 M HCOOH + 0.5 M H_2_SO_4_ (pH ≈ 0.3) 50 mV s^−1^	0.2	25.1 mA cm^−2^ (0.3 V vs. RHE)	3.7	3.3	-	-
[125]	Pd_71_In_29_	0.5 M HCOOH + 0.5 M H_2_SO_4_ (pH ≈ 0.3) 50 mV s^−1^	0.2	18.4 mA cm^−2^ (0.5 V vs. RHE)	1.2	-	-	-
[126]	Pd_54_Ag_46_ (mixed)	1 M HCOOK + 1 M KOH (pH ≈ 14.0) 50 mV s^−1^	0.2	12.2 mA cm^−2^ (0.7 V vs. RHE)	<1.0	-	-	-
	Pd_54_Ag_46_ (core-shell)	1 M HCOOK + 1 M KOH (pH ≈ 14.0) 50 mV s^−1^	0.2	31.0 mA cm^−2^ (0.7 V vs. RHE)	<1.0	-	-	-
[71]	PdCu nanoparticles	0.5 M HCOOK + 0.5 M KOH (pH ≈ 13.7) 50 mV s^−1^	0.5	84.6 mA cm^−2^ (1.2 V vs. RHE)	65.1	77.1	38.2	18.6
[118]	Pd_67_Ag_33_/Vulcan carbon	1 M HCOOK + 1 M KOH (pH ≈ 14.0) 50 mV s^−1^	0.1	6.6 mA cm^−2^ (0.6 V vs. RHE)	<0.5	<0.5	-	-
	Pd_72_Ce_28_/Vulcan carbon	1 M HCOOK + 1 M KOH (pH ≈ 14.0) 50 mV s^−1^	0.1	19.4 mA cm^−2^ (0.6 V vs. RHE)	0.8	0.7	-	-
	Pd_70_Cu_30_/Vulcan carbon	1 M HCOOK + 1 M KOH (pH ≈ 14.0) 50 mV s^−1^	0.2	4.3 mA cm^−2^ (0.6 V vs. RHE)	<0.5	<0.5	-	-
	Pd_63_Co_37_/Vulcan carbon	1 M HCOOK + 1 M KOH (pH ≈ 14.0) 50 mV s^−1^	0.2	3.5 mA cm^−2^ (0.6 V vs. RHE)	<0.5	<0.5	-	-
	Pd_65_Ni_35_/Vulcan carbon	1 M HCOOK + 1 M KOH (pH ≈ 14.0) 50 mV s^−1^	0.2	3.4 mA cm^−2^ (0.6 V vs. RHE)	<0.5	<0.5	-	-
	Pd_2.3_Co/Vulcan carbon	1 M HCOOK + 1 M KOH (pH ≈ 14.0) 50 mV s^−1^	0.3	38.0 mA cm^−2^ (0.8 V vs. RHE)	8.6	-	-	-
[120]	PdNi/Vulcan carbon	1 M HCOOK + 1 M KOH (pH ≈ 14.0) 50 mV s^−1^	0.2	74.0 mA cm^−2^ (0.8 V vs. RHE)	11.0	-	-	-
	PdNi/Ketjen carbon	1 M HCOOK + 1 M KOH (pH ≈ 14.0) 50 mV s^−1^	0.2	117.0 mA cm^−2^ (0.8 V vs. RHE)	62.0	-	-	-
[122]	Pd_3_(15%)Cu_1_(3%)/Reduced graphene oxide	0.5 M HCOOH + 0.5 M H_2_SO_4_ (pH ≈ 0.3) 50 mV s^−1^	0.1	22.9 mA cm^−2^ (0.4 V vs. RHE)	2.3	1.8	-	-
[73]	PdPt/TiO_2_	0.5 M HCOOH + 0.5 M H_2_SO_4_ (pH ≈ 0.3) 10 mV s^−1^	0.1	12.8 mA cm^−2^ (0.3 V vs. RHE)	8.3	3.7	-	-
[126]	Pd_72_Ag_19_Ni_9_ (mixed)	1 M HCOOK + 1 M KOH (pH ≈ 14.0) 50 mV s^−1^	0.2	33.7 mA cm^−2^ (0.8 V vs. RHE)	<2.0	-	-	-
[126]	Pd_60_Ag_20_Ni_20_ (alloyed)	1 M HCOOK + 1 M KOH (pH ≈ 14.0) 50 mV s^−1^	0.2	99.6 mA cm^−2^ (0.8 V vs. RHE)	18.8	-	-	-
[127]	PdAg nanotubes	0.5 M HCOOH + 0.1 M HClO_4_ (pH ≈ 1.0) 100 mV s^−1^	0.1	3.8 mA cm^−2^ (0.6 V vs. RHE)	0.7	0.5	0.6	-
[115]	Pd_2_Ag_1_ aerogel	0.5 M HCOOH + 0.5 M KOH (pH ≈ 13.7) 50 mV s^−1^	0.2	27.5 mA cm^−2^ (0.8 V vs. RHE)	2.0	1.6	-	-
[128]	Pd_50_Ag_50_ aerogel	1 M HCOOK + 1 M KOH (pH ≈ 14.0) 50 mV s^−1^	0.1	17.9 mA cm^−2^ (0.7 V vs. RHE)	0.8	-	-	-
[128]	Pd_50_Cu_50_ aerogel	1 M HCOOK + 1 M KOH (pH ≈ 14.0) 50 mV s^−1^	0.3	17.8 mA cm^−2^ (0.7 V vs. RHE)	<1.0	-	-	-
[129]	PdCu aerogel	0.5 M HCOOH + 0.5 M H_2_SO_4_ (pH ≈ 0.3) 50 mV s^−1^	0.1	174.0 mA cm^−2^ (0.5 V vs. RHE)	<10.0	<10.0	-	-
[115]	Pd_2_Ag_1_Pt_025_ aerogel	0.5 M HCOOH + 0.5 M KOH (pH ≈ 13.7) 50 mV s^−1^	0.1	60.0 mA cm^−2^ (0.7 V vs. RHE)	<2.0	3.4	-	-
[129]	B-PdCuAu nanospine assembly	0.5 M HCOOH + 0.5 M H_2_SO_4_ (pH ≈ 0.3) 50 mV s^−1^	0.1	23.2 mA cm^−2^ (0.6 V vs. RHE)	<1.0	<1.0	-	-
[130]	Pd (interstitial B)	0.5 M HCOOK + 1 M KOH (pH ≈ 14.0) 100 mv s^−1^	0.2	90 mA cm^−2^ (0.8 V vs. RHE)	5	5	-	-
[71]	PdCuPt (hierarchical zigzag-branched urchin-like superstructure)	0.5 M HCOOH + 0.5 M KOH (pH ≈ 13.7) 50 mV s^−1^	0.5	102.4 mA cm^−2^ (1.2 V vs. RHE)	75.4	101.9	75.7	22.1

^i^ Approximate pH values estimated from the acid/base concentrations, when they are not specified in the articles. ^ii^ Approximate values estimated from the experimental details and the voltammograms (forward scan) shown in the articles. ^iii^ Maximum current values taken on the reverse scan.

**Table 4:** Summary of Pt and Pd-free materials used in electrocatalytic formic acid/formate electrooxidation.

Ref.	Catalyst	Experimental Conditions ^i^	*E_onset_* (V vs. RHE) ^ii^	*j_max_* (mA cm^−2^) at *E_peak_* (V vs. RHE) ^ii^	*j* (mA cm^−2^) ^ii^ at			
					1.00 V vs. RHE	1.20 V vs. RHE	1.40 V vs. RHE	1.60 V vs. RHE
[60]	Au(111)	0.05 M HCOOH + 0.2 M NaPi (pH 3.1) 10 mV s^−1^	0.5	-	0.1	0.2	0.2	-
[58]	Au (polycrystalline bead)	0.1 M HCOOH + 0.5 M Na_2_SO_4_ (pH 3.6) 50 mV s^−1^	0.3	0.3 mA cm^−2^ (1.5 V vs. RHE)	0.3	0.3	0.3	<0.1
[54]	Au disc	0.1 M HCOOK + 0.2 M K_2_SO_4_ (pH ≈ 3.8) 50 mV s^−1^	0.7	-	0.9	1.5	-	-
[136]	Au (1 1 1)	0.1 M HCOOH + 0.1 M Py (pH = 3.4) 10 mV s^−1^	0.4	3.2 mA cm^−2^ (0.7 V vs. RHE)	0.4	0.2	-	-
[137]	Rh	0.1 M HCOOH + 1.0 M NaOH (pH ≈ 14) 5 mV s^−1^	0.2	12.5 mA cm^−2^ (0.6 V vs. RHE)	2	2	2	-
[138]	Rh	0.5 M HCOOH + 0.5M H_2_SO_4_ (pH ≈ 0) 50 mV s^−1^	0.6	0.5 mA cm^−2^ (0.8 V vs. RHE)	-	-	-	-
[139]	Ir (1 1 1)	1 M HCOOH + 0.5 M H_2_SO_4_ (pH ≈ 0) solution under He flow	0.3	-	-	-	-	-
[140]	Os/GC	1 M HCOOH 0.5 M NaClO_4_ 1 mV s^−1^	0.6	0.2 mA cm^−2^ (0.8 V vs. RHE)	-	-	-	-
[141]	IrO2	0.75 M HCOOH + 1 M H_2_SO_4_ (pH ≈ 0) 100 mV s^−1^	1.2	-	-	-	0.5	-
[63]	La_0.8_Sr_0.2_CoO_3_	2.1 M HCOOH + 0.5 M KNO_3_ (pH ≈ 1.7) 40 mV s^−1^	1.1	2.2 mA cm^−2^ (1.4 V vs. RHE)	<0	<0.1	2.2	-
[51]	NiO/rGO	0.3 M HCOOH solution (pH 3.5) 100 mV s^−1^	0.2	8.9 mA cm^−2^ (0.6 V vs. RHE)	3.2	-	-	-
[142]	WC	6 M HCOOH + 3 M H_2_SO_4_ (pH ≈ 0) ^iii^	-	60.0 mA cm^−2^ (0.3 V vs. RHE) ^iv^	-	-	-	-
[143]	WC	3 M HCOOH + 1 M H_2_SO_4_ (pH ≈ 0) ^iii^	-	0.3 μA cm^−2^ (0.3 V vs. RHE) ^v^	-	-	-	-
[144]	WC	1 M HCOOH + 0.1 M KCl (pH 5.0), 2 mV s^−1^	0.4	-	-	-	-	-
[145]	WS_2_, MoS_2_			low currents, not specified				
[53]	CoFe Prussian Blue/SnO_2_:F	0.4 M HCOOH + 1 M KNO_3_ (pH 5) 5 mV s^−1^	1.2	-	-	0.2	10.1	39.4
		1 M HCOOH + 1 M KNO_3_ (pH 5) 5 mV s^−1^	1.2	-	-	1.0	27.7	97.3
		0.4 M HCOOH + 1 M KNO_3_ (pH 13) 5 mV s^−1^	1.2	-	-	0.3	6.0	25.4
		1 M HCOOH + 1 M KNO_3_ (pH 13) 5 mV s^−1^	1.2	-	-	1.0	13.3	34
[146]	Ir/GNP	0.5M H_2_SO_4_ + 1.0M HCOOH, (pH ≈ 0) 50mV s^−1^	0.2	4.2 mA cm^−2^ (0.7 V vs. RHE)	-	-	-	-
	Ir_50_Zn_50_/GNP	0.5M H_2_SO_4_ +1.0M HCOOH, (pH ≈ 0) 50mV s^−1^	0.2	4.0 mA cm^−2^ (0.5 V vs. RHE)	-	-	-	-
[138]	Rh nano-chains	0.5M HCOOH +0.5M H_2_SO_4_ (pH ≈ 0) 50 mV s^−1^	0.4	1.9 mA cm^−2^ (0.7 V vs. RHE)	0.2	0.4	0.3	-
[147]	Au + Pin	1.0 M HCOOH + 0.5 M H_2_SO_4_ (pH ≈ 0)100 mV s^−1^	0.3	0.1 mA cm^−2^ (0.7 V vs. RHE)	0.04	-	-	-
[148]	PANI-MnO_2_	0.5M HCOOH + 0.5M H_2_SO_4_ (pH ≈ 0) 10mV s^−1^	-	-	-	-	-	-
[149]	SnO_2_ + Pin	1.0 M HCOOH + 0.5 M H_2_SO_4_ (pH ≈ 0)	0. 4	0.2 mA cm^−2^ (0.6 V vs. RHE)	-	-	-	-
[150]	Ir_1_-NC	0.5 M H_2_SO_4_ +0.5 M HCOOH (pH ≈ 0) 50 mV s^−1^	0.4	12.9 A mg^−1^ (0.7 V vs. RHE) ^vi^	3.5	2	-	-
[151]	Rh_1_-NC	0.5 M H_2_SO_4_ + 0.5 M HCOOH (pH ≈ 0) 10 mV s^−1^	0.2	16.1 A mg^−1^ (0.7 V vs. RHE) ^vi^	8	-	-	-

^i^ Approximate pH values estimated from the acid/base concentrations, when they are not specified in the articles. ^ii^ Approximate values estimated from the experimental details and the voltammograms (forward scan) shown in the articles. ^iii^ Scan rate not reported. ^iv^ Current read after 3 h, experiment carried out at 70 °C. ^v^ Current read after 20 min, experiment carried out at 70 °C. ^vi^ It is not possible to transform these values into mA cm^−2^.

**Table 5:** Proposed benchmark systems and expected electrochemical performance for formate electrooxidation using some of the most promising catalysts.

Catalyst Properties		Solution Conditions				Expected Electrochemical Performance Parameters ^ii^					
Catalyst	Catalyst Loading (mg cm^−2^)	pH ^i^	Buffer/Electrolyte	Formate Concentration	Scan Rate (mV s^−1^)	Onset Potential (V)	Maximum Current Density (mA cm^−2^)	ECSA (cm^2^ g^−1^) (Pt or Pd)	ECSA-Normalized Maximum Current Density (mA cm^−2^) (Pt or Pd)	Mass-Normalized Maximum Current Density (A mg^−1^) (Pt or Pd)	Ref.
Pt disk	N/A	0–4.5	1 M HClO_4_–0.1 M H_2_SO_4_–0.2 M KPi	0.1 HCOOH–1 M HCOONa	20–50	0.2–0.5	1.0–2.9	-	-	N/A	[40,51,52,55]
Pt(20%)/C	-	0.3–1	0.5 M H_2_SO_4_–0.1 M HClO_4_	0.5 M HCOOH	50	0.2–0.3	13.1–13.5	-	-	-	[67,68]
Pt(20%)Bi/Vulcan carbon	0.026	1	0.1 M HClO_4_	0.25 M HCOOH	50	0.1	46.1	-	-	9.06	[69]
Pt_1_Ru_1_(40%)/Vulcan carbon	1.6	~0	1 M H_2_SO_4_	1 M HCOOH	20	0.3	145	-	-	0.34	[70]
Pt(2.6 at.%)AuCu	-	1	0.1 M HClO_4_	2 M HCOOH	50	0.2	-	249	162.1 (at 1.3 V)	40.3 (at 1.3 V)	[80]
Pd disk	N/A	0.3	0.5 M H_2_SO_4_	1 M HCOOH	50–70	~0.2	~3	-	-	N/A	[30,110]
Pd(40%)/Vulcan carbon	1.6	~0	1 M H_2_SO_4_	1 M HCOOH	20	0.1	140	-	-	0.22	[70]
		14	1 M KOH	1 M HCOOK	20	0.2	102	-	-	0.16	
PdNi/Ketjen carbon	0.08	14	1 M KOH	1 M HCOOK	50	0.2	117	54	14	7.8	[120]
Nanoporous Pd (AlPd dealloyed)	0.88	0.3	0.5 M H_2_SO_4_	0.5 M HCOOH	10	0.1	232	230,000	1.1	0.262	[114]
Au disk	N/A	3.8	0.2 M K_2_SO_4_	0.1 M HCOOK	50	0.7	1.5 (at 1.2 V)	-	-	N/A	[54]
CoFe Prussian Blue/SnO_2_:F	0.3 mg CoFe-PB cm^−2^	5	1 M KNO_3_	1M HCOOH	5	1.2	97.3 (at 1.6 V)	--	--	--	[53]
		13	1 M KNO_3_	1M HCOOH	5	1.2	34.0 (at 1.6 V)	-	-	-	
Rh_1_-NC	4	~0	0.5 M H_2_SO_4_	0.5 M HCOOH	10	0.2	-	-	-	16.1	[151]

^i^ Approximate pH values estimated from the acid/base concentrations, when they are not specified in the articles. ^ii^ Approximate values estimated from the experimental details and the voltammograms (forward scan) shown in the articles.

## Data Availability

Not Applicable.

## Sample Availability

Samples of the compounds are not available from the authors.

## References

[1] Jiang S.P., Li Q. (2021). Introduction to Fuel Cells.

[2] U.S. Department of Energy (2020). DOE–Hydrogen and Fuel Cell Program. www.hydrogen.energy.gov.

[3] Ogungbemi E., Wilberforce T., Ijaodola O., Thompson J., Olabi A.G. (2021). Review of operating condition, design parameters and material properties for proton exchange membrane fuel cells. Int. J. Energy Res..

[4] Baharuddin N.A., Wan Yusoff W.N.A., Abd Aziz A.J., Mohd Tahir N.N. Hydrogen fuel cells for sustainable energy: Development and progress in selected developed countries. Proceedings of the International Postgraduate Conference on Mechanical Engineering (IPCME 2021).

[5] Shaari N., Kamarudin S.K., Bahru R., Osman S.H., Md Ishak N.A.I. (2021). Progress and challenges: Review for direct liquid fuel cell. Int. J. Energy Res..

[6] Ma Z., Legrand U., Pahija E., Tavares J.R., Boffito D.C. (2021). From CO_2_ to Formic Acid Fuel Cells. Ind. Eng. Chem. Res..

[7] Pan H., Heagy M.D. (2020). Photons to formate—A review on photocatalytic reduction of CO_2_ to formic acid. Nanomaterials.

[8] Mardini N., Bicer Y. (2021). Direct synthesis of formic acid as hydrogen carrierfrom CO_2_ for cleaner power generation throughdirect formic acid fuel cell. Int. J. Hydrogen Energy.

[9] Vo T., Purohit K., Nguyen C., Biggs B., Mayoral S., Haan J. (2015). Formate: An Energy Storage and Transport Bridge between Carbon Dioxide and a Formate Fuel Cell in a Single Device. ChemSusChem.

[10] Lu X., Wu Y., Yuan X., Wang H. (2019). An Integrated CO_2_ Electrolyzer and Formate Fuel Cell Enabled by a Reversibly Restructuring Pb–Pd Bimetallic Catalyst. Angew. Chem. Int. Ed..

[11] Xiang H., Miller H.A., Bellini M., Christensen H., Scott K., Rasul S., Yu E.H. (2019). Production of formate by CO_2_ electrochemical reduction and its application in energy storage. Sustain. Energy Fuels.

[12] Eppinger J., Huang K.W. (2017). Formic Acid as a Hydrogen Energy Carrier. ACS Energy Lett..

[13] An L., Chen R. (2016). Direct formate fuel cells: A review. J. Power Source.

[14] Li Y., Li Q., Wang H., Zhang L., Wilkinson D., Zhang J. (2019). Recent Progresses in Oxygen Reduction Reaction Electrocatalysts for Electrochemical Energy Applications. Electrochem. Energy Rev..

[15] Finkelstein D.A., Kirtland J.D., Mota N.D., Stroock A.D., Abruña H.D. (2011). Alternative Oxidants for High-Power Fuel Cells Studied by Rotating Disk Electrode (RDE) Voltammetry at Pt, Au, and Glassy Carbon Electrodes. J. Phys. Chem. C..

[16] Noyes A.A., Garner C.S. (2002). Strong Oxidizing Agents in Nitric Acid Solution. I. Oxidation Potential of Cerous—Ceric Salts. J. Am. Chem. Soc..

[17] Nair V., Deepthi A. (2007). Cerium(IV) ammonium nitrate—A versatile single-electron oxidant. Chem. Rev..

[18] Vanýsek P. (1978). Table of Standard Electrode Potentials. CRC Handbook of Chemistry and Physics.

[19] Mota N., Finkelstein D., Kirtland J., Rodriguez C., Stroock A., Abruña H. (2012). Membraneless, Room-Temperature, Direct Borohydride/Cerium Fuel Cell with Power Density of Over 0.25 W/cm^2^. J. Am. Chem. Soc..

[20] Finkelstein D.A., Abruña H.D. (2017). Rediscovering Cr_2_O_7_^2−^, an Oxidant with Unrivaled Power and Energy Density, for Affordable, Next-Generation Energy Storage and Conversion. ACS Energy Lett..

[21] Li Y., He Y., Yang W. (2015). A high-performance direct formate-peroxide fuel cell with palladium–gold alloy coated foam electrodes. J. Power Source.

[22] Li Y., Wu H., He Y., Liu Y., Jin L. (2015). Performance of direct formate-peroxide fuel cells. J. Power Source.

[23] Han L., González-Cobos J., Sánchez-Molina I., Giancola S., Folkmann S., Vidal-Ferrán A., Galán-Mascarós J.R. (2020). A low temperature aqueous formate fuel cell using cobalt hexacyanoferrate as a non-noble metal oxidation catalyst. Sustain. Energy Fuels.

[24] Singh A.K., Singh S., Kumar A. (2015). Hydrogen energy future with formic acid: A renewable chemical hydrogen storage system. Catal. Sci. Technol..

[25] Yuan X.Z., Wang H. (2008). PEM Fuel Cell Electrocatalysts and Catalyst Layers: Fundamentals and Applications.

[26] Ferrin P., Nilekar A.U., Greeley J., Mavrikakis M., Rossmeisl J. (2008). Reactivity descriptors for direct methanol fuel cell anode catalysts. Surf. Sci..

[27] Rejal S.Z., Masdar M.S., Kamarudin S.K. (2014). A parametric study of the direct formic acid fuel cell (DFAFC) performance and fuel crossover. Int. J. Hydrogen Energy.

[28] Yu X., Pickup P.G. (2008). Recent advances in direct formic acid fuel cells (DFAFC). J. Power Source.

[29] Davis D.G. (1960). The effect of platinum oxide films on reaction kinetics at platinum electrodes. Talanta.

[30] Capon A., Parsons R. (1973). The oxidation of formic acid on noble metal electrodes. II. A comparison of the behaviour of pure electrodes. J. Electroanal. Chem..

[31] Fang Z., Chen W. (2021). Recent advances in formic acid electro-oxidation: From the fundamental mechanism to electrocatalysts. Nanoscale Adv..

[32] Shen T., Zhang J., Chen K., Deng S., Wang D. (2020). Recent Progress of Palladium-Based Electrocatalysts for the FormicAcid Oxidation Reaction. Energy Fuels.

[33] Bligaard T., Bullock R.M., Campbell C.T., Chen J.G., Gates B.C., Gorte R.J., Jones C.W., Jones W.D., Kitchin J.R., Scott S.L. (2016). Toward Benchmarking in Catalysis Science: Best Practices, Challenges, and Opportunities. ACS Catal..

[34] Wei C., Rao R., Peng J., Huang B., Stephens I., Risch M., Xu Z., Shao-Horn Y. (2019). Recommended Practices and Benchmark Activity for Hydrogen and Oxygen Electrocatalysis in Water Splitting and Fuel Cells. Adv. Mater..

[35] Buck R.P., Griffith L.R. (1962). Voltammetric and Chronopotentiometric Study of the Anodic Oxidation of Methanol, Formaldehyde, and Formic Acid. J. Electrochem. Soc..

[36] Kutschker A., Vielstich W. (1963). Zum mechanismus der eletrochemischen ameisensäureoxidation in saurem leitelektrolyten. Electrochim. Acta.

[37] Breiter M.W. (1963). Anodic oxidation of formic acid on platinum-I. Adsorption of formic acid, oxygen, and hydrogen in perchloric acid solutions. Electrochim. Acta.

[38] Juliard A.L., Shalit H. (1963). Application of Cyclic Voltammetry to the Kinetic Study of Electro-Oxidation of Organic Compounds. J. Electrochem. Soc..

[39] Schmidt H., Vielstich W. (1966). Einfluß von Edelmetall-Mischkatalysatoren auf die anodische Oxydation von Methanol und Formiat. Fresenius’ Z. Anal. Chem..

[40] John J., Wang H., Rus E.D., Abruña H.D. (2012). Mechanistic studies of formate oxidation on platinum in alkaline medium. J. Phys. Chem. C.

[41] Jerkiewicz G. (2010). Electrochemical Hydrogen Adsorption and Absorption. Part 1: Under-potential Deposition of Hydrogen. Electrocatalysis.

[42] Spendelow J.S., Goodpaster J.D., Kenis P.J.A., Wieckowski A. (2006). Mechanism of CO oxidation on Pt(111) in alkaline media. J. Phys. Chem. B.

[43] Couto A., Rincón A., Pérez M.C., Gutiérrez C. (2001). Adsorption and electrooxidation of carbon monoxide on polycrystalline platinum at pH 0.3–13. Electrochim. Acta.

[44] Marković N.M., Sarraf S.T., Gasteiger H.A., Ross P.N. (1996). Hydrogen electrochemistry on platinum low-index single-crystal surfaces in alkaline solution. J. Chem. Soc. Faraday Trans..

[45] García G., Koper M.T.M. (2008). Stripping voltammetry of carbon monoxide oxidation on stepped platinum single-crystal electrodes in alkaline solution. Phys. Chem. Chem. Phys..

[46] Joo J., Uchida T., Cuesta A., Koper M.T.M., Osawa M. (2014). The effect of pH on the electrocatalytic oxidation of formic acid/formate on platinum: A mechanistic study by surface-enhanced infrared spectroscopy coupled with cyclic voltammetry. Electrochim. Acta.

[47] Miki A., Ye S., Osawa M. (2002). Surface-enhanced IR absorption on platinum nanoparticles: An application to real-time monitoring of electrocatalytic reactions. Chem. Commun..

[48] Samjeské G., Osawa M. (2005). Current oscillations during formic acid oxidation on a Pt electrode: Insight into the mechanism by time-resolved IR spectroscopy. Angew. Chem. Int. Ed..

[49] Chen Y.X., Heinen M., Jusys Z., Behm R.J. (2006). Kinetics and mechanism of the electrooxidation of formic acid–Spectroelectrochemical studies in a flow cell. Angew. Chem. Int. Ed..

[50] Chen Y.X., Heinen M., Jusys Z., Behm R.J. (2006). Bridge-bonded formate: Active intermediate or spectator species in formic acid oxidation on a Pt film electrode?. Langmuir.

[51] Kumar M.K., Jha N.S., Mohan S., Jha S.K. (2014). Reduced graphene oxide-supported nickel oxide catalyst with improved CO tolerance for formic acid electrooxidation. Int. J. Hydrogen Energy.

[52] Yang Z., Wang Y., Dong T., Yuan X., Lv L., Wei X., Wang J. (2017). Formate: A possible replacement for formic acid in fuel cells. Aust. J. Chem..

[53] Han L., González-Cobos J., Sánchez-Molina I., Giancola S., Folkman S., Tang P., Heggen M., Dunin-Borkowski R., Arbiol J., Giménez S. (2020). Cobalt Hexacyanoferrate as a Selective and High Current Density Formate Oxidation Electrocatalyst. ACS Appl. Energy Mater..

[54] Jeong S., Shin W. (2016). Triple-pulse Method for Monitoring Formate in CO_2_ Conversion Process. Electroanalysis.

[55] Perales-Rondón J.V., Brimaud S., Solla-Gullón J., Herrero E., Jürgen-Behm R., Feliu J. (2015). Further Insights into the Formic Acid Oxidation Mechanism on Platinum: pH and Anion Adsorption Effects. Electrochim. Acta.

[56] Joo J., Choun M., Jeong J., Lee J. (2015). Influence of Solution pH on Pt Anode Catalyst in Direct Formic Acid Fuel Cells. ACS Catal..

[57] Okamoto H., Kon W., Mukouyama Y. (2004). Stationary Voltammogram for Oxidation of Formic Acid on Polycrystalline Platinum. J. Phys. Chem. B.

[58] Brimaud S., Solla-Gullón J., Weber I., Feliu J.M., Behm R.J. (2014). Formic Acid Electrooxidation on Noble-Metal Electrodes: Role and Mechanistic Implications of pH, Surface Structure, and Anion Adsorption. ChemElectroChem.

[59] Busó-Rogero C., Ferre-Vilaplana A., Herrero E., Feliu J.M. (2019). The role of formic acid/formate equilibria in the oxidation of formic acid on Pt(111). Electrochem. Commun..

[60] Abdelrahman A., Hermann J.M., Kibler L.A. (2017). Electrocatalytic Oxidation of Formate and Formic Acid on Platinum and Gold: Study of pH Dependence with Phosphate Buffers. Electrocatalysis.

[61] Perales-Rondón J.V., Herrero E., Feliu J.M. (2015). On the activation energy of the formic acid oxidation reaction on platinum electrodes. J. Electroanal. Chem..

[62] Habibi B., Imanzadeh H., Haghighi Shishavan Y., Amiri M. (2020). Effect of Carbon Support on the Electrocatalytic Performance of the Pt Nanoparticles Toward Oxidation of Formic Acid. Catal. Lett..

[63] Bisht A., Pentyala P., Deshpande P.A., Sharma S. (2019). La_0.80_Sr_0.20_CoO_3_ as a noble-metal-free catalyst for the direct oxidation of formic acid under zero applied potential. Electrochem. Commun..

[64] Jiang J., Scott J., Wieckowski A. (2013). Direct evidence of a triple-path mechanism of formate electrooxidation on Pt black in alkaline media at varying temperature. Part I: The electrochemical studies. Electrochim. Acta.

[65] Han S., Liu H., Bai J., Tian X., Xia B., Zeng J., Jiang J., Chen Y. (2018). Platinum-Silver Alloy Nanoballoon Nanoassemblies with Super Catalytic Activity for the Formate Electrooxidation. ACS Appl. Energy Mater..

[66] Lashkenari M., Ghorbani M., Safabakhsh M., Shahrokhi B., Fallah J., Rezaei S. (2020). Fabrication of polyaniline/SBA-15-supported platinum/cobalt nanocomposites as promising electrocatalyst for formic acid oxidation. J. Appl. Electrochem..

[67] Shi H., Liao F., Zhu W., Shao C., Shao M. (2020). Effective PtAu nanowire network catalysts with ultralow Pt content for formic acid oxidation and methanol oxidation. Int. J. Hydrogen Energy.

[68] Menshikov V., Novomlinsky I., Belenov S., Alekseenko A., Safronenko O., Guterman V. (2021). Methanol, Ethanol, and Formic Acid Oxidation on New Platinum-Containing Catalysts. Catalysts.

[69] Wang C., Yu Z., Li G., Song Q., Li G., Luo C., Yin S., Lu B., Xiao C., Xu B. (2020). Intermetallic PtBi Nanoplates with High Catalytic Activity towards Electro-oxidation of Formic Acid and Glycerol. ChemElectroChem.

[70] Yu X., Manthiram A. (2015). Catalyst-selective, scalable membraneless alkaline direct formate fuel cells. Appl. Catal. B Environ..

[71] Chen Y., Niu H., Feng Y., Wu J., Wang A., Huang H., Feng J. (2020). Three-dimensional hierarchical urchin-like PdCuPt nanoassembles with zigzag branches: A highly efficient and durable electrocatalyst for formic acid oxidation reaction. Appl. Surf. Sci..

[72] Hsieh C., Hsiao H., Tzou D., Yu P., Chen P., Jang B. (2015). Electro-oxidation of methanol and formic acid on platinum nanoparticles with different oxidation levels. Mater. Chem. Phys..

[73] Pisarek M., Kedzierzawski P., Andrzejczuk M., Holdynsky M., Mikolajkzuk-Zichora A., Borodzinsky A., Janik-Czachor M. (2020). TiO_2_ nanotubes with pt and pd nanoparticles as catalysts for electro-oxidation of formic acid. Materials.

[74] Liang Z., Song L., Elnabawy A., Marinkovic M., Mavrikakis M., Adzic R. (2020). Platinum and Palladium Monolayer Electrocatalysts for Formic Acid Oxidation. Top. Catal..

[75] Adić R.R., Spasojević M.D., Despić A.R. (1978). Electrocatalysis by foreign metal monolayers. Oxidation of formic acid on palladium. J. Electroanal. Chem..

[76] Chen W., Xu L.P., Chen S. (2009). Enhanced electrocatalytic oxidation of formic acid by platinum deposition on ruthenium nanoparticle surfaces. J. Electroanal. Chem..

[77] Kormányos A., Speck F.D., Mayrhofer K.J.J., Cherevko S. (2020). Influence of Fuels and pH on the Dissolution Stability of Bifunctional PtRu/C Alloy Electrocatalysts. ACS Catal..

[78] Park I.S., Lee K.S., Choi J.H., Park H.Y., Sung Y.E. (2007). Surface Structure of Pt-Modified Au Nanoparticles and Electrocatalytic Activity in Formic Acid Electro-Oxidation. J. Phys. Chem. C.

[79] Chen W., Kim J., Sun S., Chen S. (2006). Electro-oxidation of formic acid catalyzed by FePt nanoparticles. Phys. Chem. Chem. Phys..

[80] Xie Y., Dimitrov N. (2020). Ultralow Pt loading nanoporous Au-Cu-Pt thin film as highly active and durable catalyst for formic acid oxidation. Appl. Catal. B Environ..

[81] Ferre-Vilaplana A., Perales-Rondón J.V., Buso-Rogero C., Feliu J.M., Herrero E. (2017). Formic acid oxidation on platinum electrodes: A detailed mechanism supported by experiments and calculations on well-defined surfaces. J. Mater. Chem. A.

[82] Aslam N.M., Masdar M.S., Kamarudin S.K., Daud W.R.W. (2012). Overview on Direct Formic Acid Fuel Cells (DFAFCs) as an Energy Sources. Apcbee Procedia.

[83] Nogalska A., Navarro A.B., Garcia-Valls R. (2020). MEA preparation for direct formate/formic acid fuel cell—Comparison of palladium black and palladium supported on activated carbon performance on power generation in passive fuel cell. Membranes.

[84] Petrii O.A. (2008). Pt-Ru electrocatalysts for fuel cells: A representative review. J. Solid State Electrochem..

[85] Marković N., Gasteiger H., Ross P., Jiang X., Villegas I., Weaver M. (1995). Electro-oxidation mechanisms of methanol and formic acid on Pt-Ru alloy surfaces. Electrochim. Acta.

[86] Gasteiger H.A., Marković N., Ross P.N., Cairns E.J. (1994). Electro-oxidation of small organic molecules on well-characterized Pt-Ru alloys. Electrochim. Acta.

[87] Neurock M., Janik M., Wieckowski A. (2008). A first principles comparison of the mechanism and site requirements for the electrocatalytic oxidation of methanol and formic acid over Pt. Faraday Discuss..

[88] Schalenbach M., Kasian O., Ledendecker M., Speck F., Mingers A., Mayrhofer K., Cherevko S. (2018). The Electrochemical Dissolution of Noble Metals in Alkaline Media. Electrocatalysis.

[89] Cherevko S., Zeradjanin A.R., Keeley G.P., Mayrhofer K.J.J. (2014). A Comparative Study on Gold and Platinum Dissolution in Acidic and Alkaline Media. J. Electrochem. Soc..

[90] da Silva S.G., Silva J.C.M., Buzzo G.S., Neto A.O., Assumpção M.H.M.T. (2016). Use of PtAu/C electrocatalysts toward formate oxidation: Electrochemical and fuel cell considerations. Mater. Renew. Sustain. Energy.

[91] Capon A., Parsons R. (1975). The oxidation of formic acid at noble metal electrodes Part 4. Platinum + palladium alloys. J. Electroanal. Chem..

[92] Waszczuk P., Crown A., Mitrovski S., Wieckowski A. (2010). Methanol and formic acid oxidation on ad-metal modified electrodes. Handbook of Fuel Cells.

[93] Watanabe M., Horiuchi M., Motoo S. (1988). Electrocatalysis by ad-atoms. Part XXIII. Design of platinum ad-electrodes for formic acid fuel cells with ad-atoms of the IVth and the Vth groups. J. Electroanal. Chem..

[94] Motoo S., Watanabe M. (1976). Electrocatalysis by Sn and Ge AD-atoms. J. Electroanal. Chem..

[95] Adžić R.R., Simić D.N., Despić A.R., Dražić D.M. (1975). Electrocatalysis by foreign metal monolayers: Oxidation of formic acid on platinum. J. Electroanal. Chem. Interfac. Electrochem..

[96] Kwon Y., Birdja Y., Spanos I., Rodriguez P., Koper M.T.M. (2012). Highly selective electro-oxidation of glycerol to dihydroxyacetone on platinum in the presence of bismuth. ACS Catal..

[97] González-Cobos J., Baranton S., Coutanceau C. (2016). A Systematic in Situ Infrared Study of the Electrooxidation of C3 Alcohols on Carbon-Supported Pt and Pt-Bi Catalysts. J. Phys. Chem. C.

[98] Tusi M.M., Polanco N.S.O., Da Silva S.G., Spinacé E.V., Neto A.O. (2011). The high activity of PtBi/C electrocatalysts for ethanol electro-oxidation in alkaline medium. Electrochem. Commun..

[99] Joo J., Uchida T., Cuesta A., Koper M.T.M., Osawa M. (2013). Importance of acid-base equilibrium in electrocatalytic oxidation of formic acid on platinum. J. Am. Chem. Soc..

[100] Zhang M., Chen W., Wei Z., Xu M., He Z., Cai J., Chen Y., Santos E. (2021). Mechanistic Implication of the pH Effect and H/D Kinetic Isotope Effect on HCOOH/HCOO–Oxidation at Pt Electrodes: A Study by Computer Simulation. ACS Catal..

[101] Haan J.L., Masel R.I. (2009). The influence of solution pH on rates of an electrocatalytic reaction: Formic acid electrooxidation on platinum and palladium. Electrochim. Acta.

[102] Rees N.V., Compton R.G. (2011). Sustainable energy: A review of formic acid electrochemical fuel cells. J. Solid State Electrochem..

[103] Beletskaya I.P. (1983). The cross-coupling reactions of organic halides with organic derivatives of tin, mercury and copper catalyzed by palladium. J. Organomet. Chem..

[104] Wolfe J.P., Li J.J. (2007). An introduction to palladium catalysis. Tetrahedron Organic Chemistry Series.

[105] Conway B.E., Bockris J.O. (1956). The d-Band Character of Metals and the Rate and Mechanism of the Electrolytic Hydrogen Evolution Reaction. Nature.

[106] Pentland N., Bockris J.O., Sheldon E. (1957). Hydrogen Evolution Reaction on Copper, Gold, Molybdenum, Palladium, Rhodium, and Iron. J. Electrochem. Soc..

[107] Savadogo O., Lee K., Oishi K., Mitsushima S., Kamiya N., Ota K. (2004). New palladium alloys catalyst for the oxygen reduction reaction in an acid medium. Electrochem. Commun..

[108] Wang J.-Y., Zhang H.-X., Jiang K., Cai W.-B. (2011). From HCOOH to CO at Pd Electrodes: A Surface-Enhanced Infrared Spectroscopy Study. J. Am. Chem. Soc..

[109] Arenz M., Stamenkovic V., Schmidt T., Wandelt K., Ross P., Markovic N. (2003). The electro-oxidation of formic acid on Pt-Pd single crystal bimetallic surfaces. Phys. Chem. Chem. Phys..

[110] Solis V., Iwasita T., Pavese A., Vielstich W. (1988). Investigation of formic acid oxidation on palladium in acidic solutions by on-line mass spectroscopy. J. Electroanal. Chem..

[111] Hoshi N., Kida K., Nakamura M., Nakada M., Osada K. (2006). Structural Effects of Electrochemical Oxidation of Formic Acid on Single Crystal Electrodes of Palladium. J. Phys. Chem B.

[112] Choi S., Herron J., Scaranto J., Huang H., Wang Y., Xia X., Lv T., Park J., Peng H., Mavrikakis M. (2015). A Comprehensive Study of Formic Acid Oxidation on Palladium Nanocrystals with Different Types of Facets and Twin Defects. ChemCatChem.

[113] Wang H., Qian X., Lui S., Yin S., Xu Y., Li X., Wang Z., Wang L. (2020). Boron-Doped PdCuAu Nanospine Assembly as an Efficient Electrocatalyst toward Formic Acid Oxidation. Chem. Eur. J..

[114] Wang X., Wang W., Qi Z., Zhao C., Ji H., Zhang Z. (2009). High catalytic activity of ultrafine nanoporous palladium for electro-oxidation of methanol, ethanol, and formic acid. Electrochem. Commun..

[115] Wang J., Chen F., Jin Y., Guo L., Gong X., Wang X., Johnston R. (2019). In situ high-potential-driven surface restructuring of ternary AgPd-Ptdilute aerogels with record-high performance improvement for formate oxidation electrocatalysis. Nanoscale.

[116] Hong S., Hwang H., Kim J.W., Lee J. (2018). The Effect of Synthesis Temperature on Pd-H Catalyst Structure for Alkaline Direct Formate Fuel Cells. ECS Trans..

[117] Galvan V., Glass D.E., Baxter A.F., Surya Prakash G.K. (2019). Reduced Graphene Oxide Supported Palladium Nanoparticles for Enhanced Electrocatalytic Activity toward Formate Electrooxidation in an Alkaline Medium. ACS Appl. Energy Mater..

[118] Tang Q., Chen F., Jin T., Guo L., Wang Q., Liu H. (2019). Alloying in inverse CeO_2_/Pd nanoparticles to enhance the electrocatalytic activity for the formate oxidation reaction. J. Mater. Chem. A.

[119] Choun M., Ham K., Shin D., Lee J.K., Lee J. (2017). Catalytically active highly metallic palladium on carbon support for oxidation of HCOO^−^. Catal. Today.

[120] Sankar S., Anilkumar G.M., Tamaki T., Yamaguchi T. (2019). Binary Pd–Ni Nanoalloy Particles over Carbon Support with Superior Alkaline Formate Fuel Electrooxidation Performance. ChemCatChem.

[121] Ren J., Zhang J., Yang C., Yang Y., Zhang Y., Yang F., Ma R., Yang L., He H., Huang H. (2020). Pd nanocrystals anchored on 3D hybrid architectures constructed from nitrogen-doped graphene and low-defect carbon nanotube as high-performance multifunctional electrocatalysts for formic acid and methanol oxidation. Mater. Today Energy.

[122] Wang H., Chen H., Wang H., Ou C., Li R., Liu H. (2020). Synthesis of ultrafine low loading Pd–Cu alloy catalysts supported on graphene with excellent electrocatalytic performance for formic acid oxidation. Int. J. Hydrogen Energy.

[123] Yépez O., Scharifker B.R. (2002). Oxidation of formate on hydrogen-loaded palladium. Int. J. Hydrogen Energy.

[124] Gharib A., Arab A. (2020). Electrodeposited Pd, PdCd, and PdBi nanostructures: Preparation, characterization, corrosion behavior, and their electrocatalytic activities for formic acid oxidation. J. Electroanal. Chem..

[125] Hwang E., Park H., Kim H., Ahn S.H., Kim S.K. (2017). Electrochemically Fabricated Pd–In Catalysts for Carbon Dioxide-Formate/Formic Acid Inter-Conversion. Bull. Korean Chem. Soc..

[126] Wang Q., Chen F., Tang Q., Guo L., Gebremarian T., Jin T., Liu H., Kou B., Li Z., Bian W. (2020). Transition from core-shell to janus segregation pattern in AgPd nanoalloy by Ni doping for the formate oxidation. Appl. Catal. B Env..

[127] Lu Y., Chen W. (2010). Nanoneedle-covered Pd-Ag nanotubes: High electrocatalytic activity for formic acid oxidation. J. Phys. Chem. C.

[128] Wang Q., Chen F., Guo L., Jin T., Liu H., Wang X., Gong X., Liu Y. (2019). Nanoalloying effects on the catalytic activity of the formate oxidation reaction over AgPd and AgCuPd aerogels. J. Mater. Chem. A.

[129] Douk A., Farsadrooh M., Damanigol F., Moghaddam A., Saravani H., Noroozifar N. (2018). Porous three-dimensional network of Pd-Cu aerogel toward formic acid oxidation. RSC Adv..

[130] Hong S., Chung S., Park J., Hwang J., Lee C., Uhm S., Bong S., Lee J. (2021). Contribution of Interstitial Boron in a Boron-Incorporated Palladium Catalyst Toward Formate Oxidation in an Alkaline Direct Formate Fuel Cell. ACS Catal..

[131] Capon A., Parsons R. (1973). The oxidation of formic acid at noble metal electrodes Part I. Review of Previous Work. Electroanal. Chem. Interfaclal Electrochem..

[132] Capon A., Parsons R. (1973). The oxidation of formic acid at noble metal electrodes Part III. Intermediates and mechanism on platinum electrodes. Electroanal. Chem. Interfac. Electrochem..

[133] Sankar S., Anilkumar G.M., Tamaki T., Yamaguchi T. (2018). Cobalt-Modified Palladium Bimetallic Catalyst: A Multifunctional Electrocatalyst with Enhanced Efficiency and Stability toward the Oxidation of Ethanol and Formate in Alkaline Medium. ACS Appl. Energy Mater..

[134] Xi Z., Li J., Su D., Muzzio M., Yi C., Li Q., Sun S. (2017). Stabilizing CuPd Nanoparticles via CuPd Coupling to WO_2.72_ Nanorods in Electrochemical Oxidation of Formic Acid. J. Am. Chem. Soc..

[135] Rettenmaier C., Arán-Ais R., Timoshenko J., Rizo R., Jeon H., Külh S., Chee S., Bergmann A., Cuenya B. (2020). Enhanced Formic Acid Oxidation over SnO_2_-decorated Pd Nanocubes. ACS Catal..

[136] Hermann J.M., Mattausch Y., Weiß A., Jacob T., Kibler L.A. (2018). Enhanced Electrocatalytic Oxidation of Formic Acid on Au(111) in the Presence of Pyridine. J. Electrochem. Soc..

[137] Burke L.D., O’Dwyer K.J. (1990). Application of the hydrous oxide mediation model of electrocatalysis to reactions at noble metal anodes in base-I.; Pt, Pd and Rh. Electrochim. Acta.

[138] Sathe B.R., Balan B.K., Pillai V.K. (2011). Enhanced electrocatalytic performance of interconnected Rh nano-chains towards formic acid oxidation. Energy Environ. Sci..

[139] Motoo S., Furuya N. (1986). Electrochemistry of iridium single crystal surfaces. Part, I. Structural effect on formic acid oxidation and poison formation on Ir(111), (100) and (110). J. Electroanal. Chem..

[140] Orozco G., Gutiérrez C. (2000). Adsorption and electro-oxidation of carbon monoxide, methanol, ethanol and formic acid on osmium electrodeposited on glassy carbon. J. Electroanal. Chem..

[141] Ouattara L., Fierro S., Frey O., Koudelka M., Comninellis C. (2009). Electrochemical comparison of IrO_2_ prepared by anodic oxidation of pure iridium and IrO_2_ prepared by thermal decomposition of H_2_IrCl_6_ precursor solution. J. Appl. Electrochem..

[142] Lindner V.E., Vitzthum I.L.G., Baresel V.D., Gellcrt W., Heidemeyer J. (1971). Bindungsisomerie bei Sulfinato-Komplexen von Obergangsmetallen Wolframcarbid als Anodenmaterial fur Brenn-stoffzellen. Angew. Chem..

[143] Palanker V.S., Gajyev R.A., Sokolsky D.V. (1977). On adsorption and electro-oxidation of some compounds on tungsten carbide; their effect on hydrogen electro-oxidation. Electrochim. Acta.

[144] Rosenbaum M., Zhao F., Quaas M., Wulff H., Schröder U., Scholz F. (2007). Evaluation of catalytic properties of tungsten carbide for the anode of microbial fuel cells. Appl. Catal. B Environ..

[145] Böhm H. (1970). New non-noble metal anode catalysts for acid fuel cells. Nature.

[146] Ahmad K.N., Noor Azam A.M.I., Isahak W.N.R.W., Mohd Zainoodin A., Masdar M.S. (2020). Improving the electrocatalytic activity for formic acid oxidation of bimetallic Ir-Zn nanoparticles decorated on graphene nanoplatelets. Mater. Res. Express.

[147] Kumar A., Joshi L., Prakash R. (2013). Electrocatalytic performance of interfacially synthesized Au-polyindole composite toward formic acid oxidation. Ind. Eng. Chem. Res..

[148] Prakash G.K.S., Suresh P., Viva F., Olah G.A. (2008). Novel single step electrochemical route to γ-MnO_2_ nanoparticle-coated polyaniline nanofibers: Thermal stability and formic acid oxidation on the resulting nanocomposites. J. Power Source.

[149] Kumar A., Pandey A.C., Prakash R. (2012). Electro-oxidation of formic acid using polyindole-SnO_2_ nanocomposite. Catal. Sci. Technol..

[150] Li Z., Chen Y., Ji S., Tang Y., Chen W., Li A., Zhao J., Xiong W., Wu Y., Gong Y. (2020). Iridium single-atom catalyst on nitrogen-doped carbon for formic acid oxidation synthesized using a general host–guest strategy. Nat. Chem..

[151] Xiong Y., Dong J., Huang Z., Xin P., Chen W., Wang Y., Li Z., Yin Z., Xin W., Zhuang Z. (2020). Single-atom Rh/N-doped carbon electrocatalyst for formic acid oxidation. Nat. Nanotechnol..

[152] Crépy G., Lamy C., Maximovitch S. (1974). Oxydation de l’acide formique sur électrode d’or. J. Electroanal. Chem..

[153] Cuesta A., Cabello G., Hartl F., Escudero-Escribano M., Vazquez-Dominguez C., Kibler L., Osawa M., Gutierrez C. (2013). Electrooxidation of formic acid on gold: An ATR-SEIRAS study of the role of adsorbed formate. Catal. Today.

[154] Kibler L.A., Al-Shakran M. (2016). Adsorption of Formate on Au(111) in Acid Solution: Relevance for Electro-Oxidation of Formic Acid. J. Phys. Chem. C.

[155] Fierro S., Ouattara L., Calderon E., Passas-Lagos E., Baltruschat H., Comninellis C. (2009). Investigation of formic acid oxidation on Ti/IrO_2_ electrodes. Electrochim. Acta.

[156] Ma C.A., Zhang W.K., Cheng D.H., Zhou B.X. (2002). Preparation and electrocatalytic properties of tungsten carbide electrocatalysts. Trans. Nonferrous Met. Soc. China.

[157] Baresel D., Gellert W., Heidemeyer J., Scharner P. (1971). Wolframcarbid als Anodenmaterial für Brennstoffzellen. Angew. Chem..

[158] Kim D., Cargnello M. (2020). Formic acid oxidation boosted by Rh single atoms. Nat. Nanotechnol..

